# Interference with glutamate antiporter system x_c_^−^ enables post‐hypoxic long‐term potentiation in hippocampus

**DOI:** 10.1113/EP092045

**Published:** 2024-08-17

**Authors:** Bradley S. Heit, Alex Chu, Alyssa McRay, Janet E. Richmond, Charles J. Heckman, John Larson

**Affiliations:** ^1^ Department of Neuroscience and Department of Biomedical Engineering Northwestern University Chicago Illinois USA; ^2^ Department of Psychiatry University of Illinois at Chicago Chicago Illinois USA; ^3^ Department of Biological Sciences University of Illinois at Chicago Chicago Illinois USA

**Keywords:** cystine/glutamate transporter, excitotoxicity, hypoxia, long‐term potentiation, stroke

## Abstract

Our group previously showed that genetic or pharmacological inhibition of the cystine/glutamate antiporter, system x_c_^−^, mitigates excitotoxicity after anoxia by increasing latency to anoxic depolarization, thus attenuating the ischaemic core. Hypoxia, however, which prevails in the ischaemic penumbra, is a condition where neurotransmission is altered, but excitotoxicity is not triggered. The present study employed mild hypoxia to further probe ischaemia‐induced changes in neuronal responsiveness from wild‐type and xCT KO (xCT^−/−^) mice. Synaptic transmission was monitored in hippocampal slices from both genotypes before, during and after a hypoxic episode. Although wild‐type and xCT^−/−^ slices showed equal suppression of synaptic transmission during hypoxia, mutant slices exhibited a persistent potentiation upon re‐oxygenation, an effect we termed ‘post‐hypoxic long‐term potentiation (LTP)’. Blocking synaptic suppression during hypoxia by antagonizing adenosine A_1_ receptors did not preclude post‐hypoxic LTP. Further examination of the induction and expression mechanisms of this plasticity revealed that post‐hypoxic LTP was driven by NMDA receptor activation, as well as increased calcium influx, with no change in paired‐pulse facilitation. Hence, the observed phenomenon engaged similar mechanisms as classical LTP. This was a remarkable finding as theta‐burst stimulation‐induced LTP was equivalent between genotypes. Importantly, post‐hypoxic LTP was generated in wild‐type slices pretreated with system x_c_^−^ inhibitor, *S*‐4‐carboxyphenylglycine, thereby confirming the antiporter's role in this phenomenon. Collectively, these data indicate that system x_c_^−^ interference enables neuroplasticity in response to mild hypoxia, and, together with its regulation of cellular damage in the ischaemic core, suggest a role for the antiporter in post‐ischaemic recovery of the penumbra.

## Abstract (highlights)

- **What is the central question of this study?**
  Does glutamate antiporter system x_c_^−^ influence recovery in the ischemic penumbra?
- **What is the main finding and its importance?**
  Our data reveal that mild hypoxia elicits a persistent potentiation of synaptic transmission in hippocampus when system x_c_^−^ is genetically deleted or pharmacologically inhibited. This ‘post‐hypoxic long‐term potentiation (LTP)’ engages similar mechanims as classical NMDAR‐dependent LTP induced by theta‐burst stimulation (TBS). Because this neuroplasticity potentially fosters recovery in penumbral tissue, these findings portend system x_c_^−^ antagonism as a post‐stroke therapeutic intervention.

## Graphical abstract

(a) When a cerebral blood vessel becomes occluded during stroke, two distinct regions form: the ischaemic core and the ischaemic penumbra. The core suffers severe oxygen deprivation (anoxia), glutamate excitotoxicity, anoxic depolarization and rapid cell death. The penumbra, which is the outer rim of the core, experiences mild ischaemia (hypoxia) and altered synaptic signalling but retains the potential to be spared via adaptations in neuronal excitability. (b–e) Employing hippocampal slice electrophysiology, we modelled the ischaemic penumbra by subjecting wild‐type (WT) mice and system x_c_^−^ knockout (xCT^−/−^) mice to hypoxia. When compared to pre‐hypoxia baseline responses (PRE), adenosine‐mediated suppression of synaptic responses during hypoxia (HYP) was equivalent between genotypes. xCT^−/−^ responses, however, displayed potentiation in the post‐hypoxia phase (POST), which resembled LTP. This post‐hypoxic LTP was abolished by the NMDA receptor antagonist DAP5, but was unaffected by the A_1_R antagonist CPX and system x_c_^−^ inhibitor CPG. WT responses phenocopied post‐hypoxic LTP after treatment with CPG or when incubated in elevated Ca^2+^ concentrations. Collectively, these data suggest that post‐hypoxic LTP with system x_c_^−^ interference is driven by enhanced postsynaptic depolarization and (1) removal of Mg^2+^ from NMDARs which (2) allows Ca^2+^ influx, thus (3) increasing AMPA receptor insertion. Moreover, these findings reveal a potential mechanism for improved recovery in penumbral tissue when system x_c_^−^ is absent or inhibited.

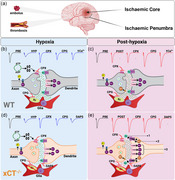

## 1 INTRODUCTION

The cystine/glutamate antiporter, system x_c_^−^ supplies 60%–80% of the tonic levels of extracellular glutamate present in the brain (Baker et al., 2002; DeBundel et al., 2011; Massie et al., 2011). System x_c_^−^ is an astrocyte‐bound (Ottestad‐Hansen et al., 2018) Na^+^‐independent transporter that imports anionic cystine in exchange for glutamate at a 1:1 molar ratio (Bannai, 1984, 1986). After transport via system x_c_^−^, intracellular cystine is converted to cysteine—the rate‐limiting substrate for glutathione (GSH) synthesis (Bannai & Tateishi, 1986; Miura et al., 1992). The antiporter therefore influences two principal physiological functions in the brain: antioxidant defence and glutamatergic neurotransmission. Not surprisingly, system x_c_^−^ has become an attractive target for studying neuronal responses to oxygen deprivation, and a growing body of research implicates the antiporter's influence in stroke aetiology. For example, prior studies have shown reduced neuronal death after ischaemic challenge in cell culture systems (Hsieh et al., 2017; Jackman et al., 2012; Soria et al., 2014; Thorn et al., 2015) as well as protection from cerebral ischaemia in vivo (Domercq et al., 2016; He & Hewett, 2022; Hsieh et al., 2017) when system x_c_^−^ is absent or inhibited.

Our group previously employed hippocampal slice electrophysiology to compare neuronal responsiveness between WT mice and transgenic mice lacking a functional system x_c_^−^ due to deletion of the system x_c_^−^ subunit xCT (xCT KO; xCT^−/−^ mice). In conditions of total oxygen deprivation (anoxia), our results revealed that brain slices with a functional system x_c_^−^ reach anoxic depolarization (AD) faster and display enhanced depolarizing waves compared to slices where the antiporter is absent or pharmacologically blocked (Heit et al., 2022). From our data we concluded that tonic glutamate supplied by system x_c_^−^ synergizes with synaptically released glutamate to exacerbate the ischaemic cascade and propagate AD. That being said, the sudden onset and rapid regenerative nature of AD may have obscured subtle, but important, antecedent differences between these two genotypes. Partial (‘mild’) hypoxia is an ischaemic condition that alters synaptic signalling but is not noxious enough to induce glutamate excitotoxicity or AD. Mild hypoxia elicits a suppression of synaptic transmission proportional to the degree of oxygen deprivation, ATP breakdown and accumulation of extracellular adenosine (Dale et al., 2000; Frenguelli et al., 2003; Heit et al., 2021; Laghi Pasini et al., 2000; Larson et al., 2014; Zetterstrom et al., 1982). As such, mild hypoxia allows us to examine energy‐dependent neuronal responsiveness during less severe ischaemic conditions, where neurotoxicity has not been evoked.

The use of anoxia and/or mild hypoxia as experimental models for ischaemia provides distinct insights into the pathogenesis of stroke. The ischaemic *core* represents tissue suffering from severe oxygen deprivation (anoxia) and fulminant neuronal death (Lipton, 1999), which is clinically defined (Nakamura et al., 2010) and diagnosed (Ayata, 2018; Hartings et al., 2017) as the region of anoxic depolarization. The *penumbra*, on the other hand, is the outer rim of the ischaemic core (Ramos‐Cabrer et al., 2011), wherein the infarct is evolving, which experiences mild ischaemia (hypoxia). These are important distinctions as the cellular processes taking place in the core versus the penumbra are vastly different and ultimately dictate the potential for therapeutic intervention. Anoxia triggers the swift collapse of electrochemical gradients and synaptic transmission, whereas hypoxia will alter synaptic signalling, but not induce glutamate excitotoxicity nor destabilize transmembrane ionic gradients (Heit et al., 2021). Although there are currently no treatments to attenuate the rapid cell death within the ischaemic core, penumbral tissue retains the potential to be spared via neuroplastic mechanisms (Li et al., 2020; Liu et al., 2019; Shen et al., 2021; Wang et al., 2021).

The present study employed mild hypoxia to further elucidate ischaemia‐induced alterations in neuronal responsiveness from WT and xCT KO (xCT^−/−^) mice. Hippocampal slices from both genotypes were prepared, incubated, subjected to 30 min hypoxia, and fully re‐oxygenated. Although WT and xCT^−/−^ slices did not differ in hypoxia‐induced synaptic suppression, mutant slices exhibited an accelerated rate of recovery and post‐hypoxic potentiation, which resembled long‐term potentiation (LTP). Experiments using CPX, an A_1_ adenosine receptor (A_1_R) antagonist, showed that this differential effect was not due to decreased adenosine release or accumulation in xCT^−/−^ slices. Contrarily, further experimentation revealed that post‐hypoxic LTP in xCT^−/−^ mice was driven by activation of NMDA receptors (NMDARs) and enhanced calcium influx with no alteration to paired‐pulse facilitation. Thus, post‐hypoxic changes in mutant mice are generated by mechanisms similar to those of classical LTP. This latter finding was remarkable as xCT^−/−^ mice showed no differences in LTP induced by theta‐burst stimulation (TBS) when compared to WT. In conjunction with our prior report (Heit et al., 2022), these data further confirm system x_c_^−^ as an important modulator of ischaemia‐induced neuronal excitability and expand the burgeoning breadth of data reporting prophylaxis with system x_c_^−^ inhibition. Absence and/or antagonism of system x_c_^−^ can both (1) delay AD during severe oxygen deprivation (anoxia), which ostensibly mitigates the ischaemic core, and (2) induce LTP after conditions of mild hypoxia, likely promoting neuroplasticity in the ischaemic penumbra.

## 2 METHODS

### 2.1 Ethical approval

All mice involved in this study were allowed ad libitum access to food and water, and cages were equipped with igloo shelters and nesting material. All experiments were terminal, wherein mice were humanely and rapidly decapitated without anaesthesia administration. All experiments complied with regulations of animal welfare protocols and were approved by the Animal Care Committee at the University of Illinois at Chicago (ACC protocol no. 20‐206) in accordance with the guidelines for euthanasia established by the American Veterinary Medical Association (AMVA). The investigators fully acknowledge the standards put forth by *Experimental Physiology* regarding the ethical care of research animals.

### 2.2 Animals

Experiments were conducted using 2‐ to 4‐month‐old male xCT knockout (xCT^−/−^) mice in our colony, bred from mice generously provided by Hideyo Sato (Yamagata University, Japan; Sato et al., 2005) or xCT^+/+^ (WT) controls. The xCT knockout mutation was generated in the C57BL/6J background and out‐crossed with C57BL/6J >10 times. xCT expression and function are completely eliminated in these xCT^−/−^ mice. Age‐matched homozygous C57BL/6J male mice were used as controls. Previous studies from our group have identified significant differences in neuronal responses to oxygen deprivation between male and female mice (Figure 10 in Heit et al., 2021); therefore, in order to decrease within‐group variance for each experiment, only male cohorts were used for the current investigation. Furthermore, we wanted our data to be comparable to the extant literature elucidating hypoxia tolerance and LTP, which almost exclusively involves males.

Hippocampal slices were prepared from WT and xCT^−/−^ mice in the conventional manner (Larson & Park, 2009; Larson et al., 1999), and the investigator (B.S.H.) was blinded to animal genotype. Briefly, mice were decapitated and the brain rapidly excised. Anaesthetic was not administered as it can alter subsequent hypoxia tolerance in slices (Bickler et al., 2005). The hippocampus was then dissected free, and slices cut on a tissue chopper at 400 µm transverse to the long axis. Slices were then maintained in an interface chamber at 34°C and continually perfused with artificial cerebrospinal fluid (ACSF) containing the following (in mM): NaCl 124, KCl 3.0, KH_2_PO_4_ 1.0, NaHCO_3_ 26, MgSO_4_ 2.0, CaCl_2_ 2.0, d‐glucose 10, and sodium l‐ascorbate 1.9 (unless specified otherwise), gassed with 95% O_2_ and 5% CO_2_. In some experiments, the concentration of calcium in the ACSF medium was manipulated. Recordings were made in the interface chamber with constant perfusion (1.0 ml min^−1^) of ACSF at 34°C with the upper surface exposed to an atmosphere of 95% O_2_ and 5% CO_2_. The gas supplied to the chamber flowed at a rate of 1 L min^−1^.

### 2.3 Electrophysiology

One stimulating electrode was utilized to evoke field potentials, which were recorded with glass electrodes filled with 2 M NaCl (1−5 MΩ). To monitor synaptic transmission, a stimulating electrode was placed in stratum radiatum of field CA1c to activate Schaffer‐commissural (SC) fibre‐evoked synaptic field potentials recorded in stratum radiatum of CA1b. Laminar profiles were used to place electrodes optimally in each slice tested. Apart from Figures 3 and 8, all experiments were paired, where two slices were compared within the same experimental conditions with one orthodromic response being recorded from each slice. We also continuously monitored all recordings at low frequency on an oscilloscope to ensure the absence of ‘anoxic depolarization’ events.

Evoked responses were amplified (100−500×), filtered (bandpass 0.1–5 kHz), digitized by microcomputer (PC), and analysed on‐line using custom software (Labview, National Instruments, Austin, TX, USA). Field excitatory postsynaptic potentials (fEPSPs) were evoked at 10 s intervals throughout the experiments. Baseline recordings were taken for at least 12 min prior to manipulations. Initial slope and peak amplitude were calculated for each fEPSP and normalized to the baseline mean in each slice. In some experiments, paired pulse facilitation (PPF) was assessed using stimuli separated by 75 ms intervals. In these cases, PPF was calculated as the percentage increase in the amplitude of the second response relative to that of the first response of the pair.

Theta‐burst stimulation (TBS) was employed as two episodes of five theta‐bursts (four pulses at 100 Hz with 200 ms between the bursts) separated by 5 s as previously described (Larson & Munkácsy, 2014). The magnitude of TBS‐induced LTP was calculated as the mean fEPSP amplitude for the final 5 min of the 60 min post‐TBS phase and quantified as a percentage of the baseline amplitude.

Specific parameters were implemented in determining which hippocampal slices would be used for experimentation. Slices deemed acceptable for measurements had to display (1) the capacity to generate an evoked potential of at least 4.0 mV in amplitude, (2) fEPSPs which maintained a half‐width ≤7.0 ms for the duration of the baseline period, and (3) PPF values between 135% and 165% for the duration of the baseline period. Furthermore, throughout the baseline period, amplitudes of individual responses did not deviate more than ±0.2 mV from the initial response captured at the start of the baseline period. This was to verify that evoked responses were stable and not ‘trending’ upward or downward prior to hypoxic intervention or drug perfusion. Any slices which did not meet these criteria were excluded from testing.

### 2.4 Hypoxia

‘Mild hypoxia’ was induced by replacing the O_2_ content of the chamber atmosphere and perfusion ACSF with a varied concentration of N_2_ (Fowler, 2003; Larson & Park, 2009) for a 30 min epoch. In these cases, the %O_2_ content refers to the proportion of the non‐CO_2_ component of the gas supply (CO_2_ was always maintained at 5%). For normoxia, the non‐CO_2_ component was 100% O_2_; for 25% O_2_, this component was 25% O_2_ and 75% N_2_ (mild hypoxia). At the end of the 30 min hypoxic phase, the suppression of synaptic transmission was calculated as the mean percentage change in fEPSP amplitude during the final 3 min of the hypoxic phase. Immediately thereafter, 100% oxygen was reinstated, and the rate of recovery was measured as the time required to attain 85% (*T*_85_) or 100% (*T*_100_) of pre‐hypoxic baseline amplitude. The magnitude of recovery, or post‐hypoxic LTP, was calculated 45 min after reinstatement of 100% O_2_. Specifically, post‐hypoxic LTP was quantified as the mean fEPSP amplitude for the final 5 min of the re‐oxygenation phase and presented as a percentage of pre‐hypoxic baseline amplitude. No slices were exposed to more than one episode of hypoxia. This is important because exposure to oxygen deprivation may inflict irreversible damage or produce a ‘preconditioning’ effect on surviving neurons (Kirino, 2002).

### 2.5 Pharmacology

All drugs were obtained from Tocris Bioscience/Bio‐Techne (Minneapolis, MN, USA). The NMDA receptor antagonist d‐2‐amino‐5‐phosphonopentanoic acid (d‐AP5) was dissolved in double distilled H_2_O at 50 mM and diluted in ACSF to a final concentration of 50 µM. The adenosine A_1_ receptor antagonist 8‐cyclopentyl‐1,3‐dipropylxanthine (CPX) was dissolved in 100% dimethyl sulfoxide at 10 mM and diluted in ACSF to a final concentration of 300 nM. The system x_c_^−^ inhibitor *S*‐4‐carboxyphenylglycine (CPG) was dissolved in NaOH (0.1 M) at 50 mM and diluted in ACSF to a final concentration of 100 µM. ‘Vehicle’ treatment used the same solution without the added drug.

### 2.6 Experimental design and statistical analyses

Data are presented as means ± standard deviation (SD), and *n* is the number of animals in the sample. Statistical analyses were performed using Prism 10.2 (GraphPad Software, Boston, MA, USA). For comparisons involving one independent variable and two groups, Student's *t‐*test (two‐tailed) was used. Slices treated in the same chamber at the same time were treated as paired samples. Mean differences were considered significant at *P *< 0.05. Comparisons involving more than one independent variable utilized the two‐ or three‐way analysis of variance (ANOVA). When ANOVA was significant, planned comparisons were conducted using Šidák's or Tukey's test. All schematic illustrations were generated using BioRender software (www.biorender.com).

## 3 RESULTS

### 3.1 WT and xCT^−/−^ mice show similar steady‐state suppression of synaptic transmission during hypoxia

Our first analysis compared WT and xCT^−/−^ mice for changes in synaptic transmission during mild hypoxia. In each run, synaptic responses were recorded from WT and xCT^−/−^ slices in the same chamber simultaneously (i.e. ‘paired experiments’). Responses were monitored for a baseline period of ≥12 min, subjected to a 30 min hypoxic episode (25% O_2_/75% N_2_), fully reoxygenated, and allowed to recover for 45 min. For the baseline period, stimulation intensity was set to evoke field excitatory postsynaptic potential (fEPSP) amplitudes ranging from 2.5 to 3.5 mV for both WT (2.960 ± 0.488 mV) and xCT^−/−^ (2.851 ± 0.298 mV) slices. Accordingly, no difference in baseline fEPSP amplitudes was observed between genotypes (paired *t*_11 _= 0.732, *P* = 0.4796. Importantly, the current input required to generate baseline responses for both WT (12.040 ± 4.302 µA) and xCT^−/−^ (10.230 ± 3.689 µA) slices also did not differ between genotypes (paired *t*_11 _= 1.393, *P* = 0.1910). These data are consistent with recent input–output analyses for young and aged xCT^−/−^ mice (Verbruggen et al., 2022).

**FIGURE 1:**
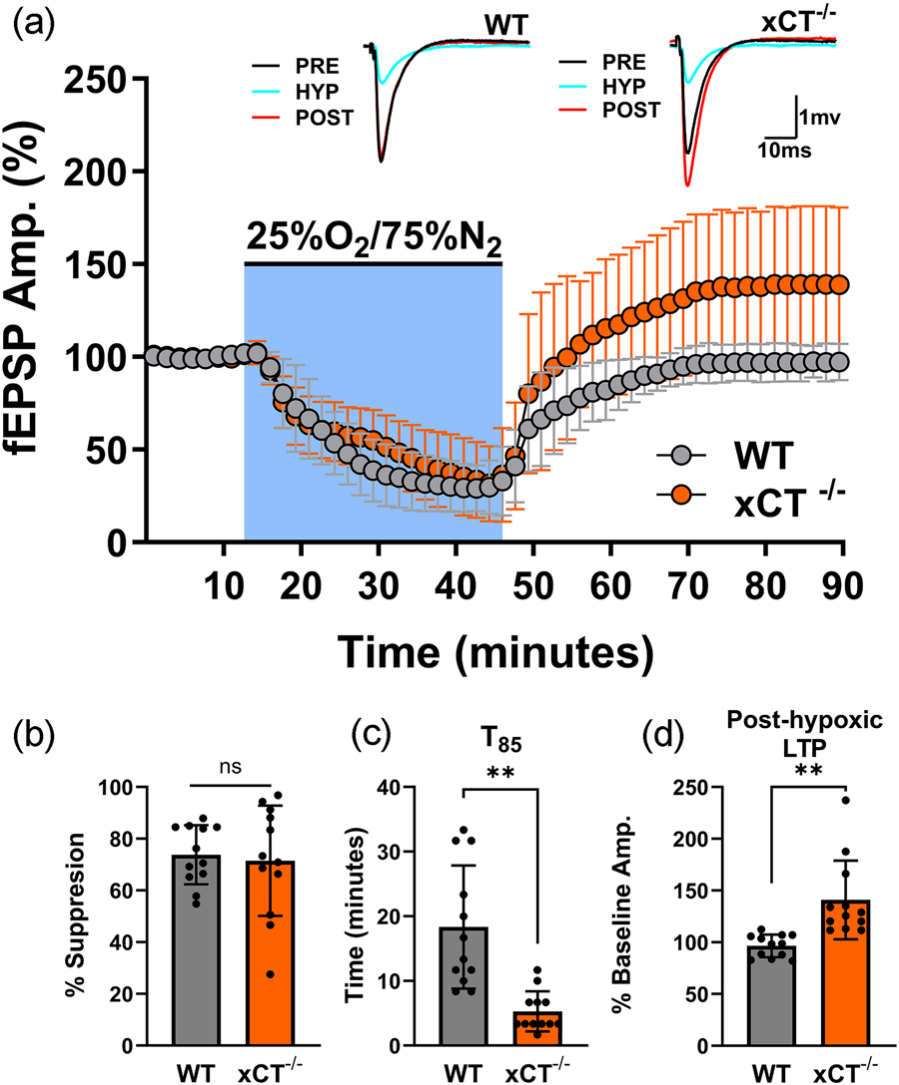
Hypoxia‐induced changes in synaptic transmission for WT and xCT^−/−^ slices. (a) Averaged data from 12 paired experiments show the effects of hypoxia on fEPSPs in WT and xCT^−/−^ slices. Each point is a mean of five consecutive trials for WT (grey) and xCT^−/−^ (orange) slices within each experiment, normalized to the pre‐hypoxic baseline mean, and averaged (mean ± SD) across experiments at each point. Hypoxia (25% O_2_/75% N_2_) was applied for 30 min (blue shaded area). Insets show averaged (*n* = 5) synaptic responses during the pre‐hypoxic baseline (PRE; immediately prior to hypoxia), hypoxic (HYP; last trials before re‐oxygenation) and post‐hypoxic (POST; 45 min after re‐oxygenation) phases for WT and xCT^−/−^ slices in a representative experiment. (b) Bar graph comparing the degree of fEPSP suppression for WT and xCT^−/−^ slices during the hypoxic episode (means ± SD). (c) Bar graph (means ± SD) comparing the time required to attain 85% of baseline amplitudes (*T*_85_) after re‐oxygenation for WT and xCT^−/−^ slices. (d) Bar graph (means ± SD) comparing magnitude of post‐hypoxic LTP for WT and xCT^−/−^ slices (data are expressed as percentage of the pre‐hypoxic baseline mean).

Figure 1 summarizes the results from paired hypoxia experiments comparing WT and xCT^−/−^ slices. Hypoxia resulted in the suppression of synaptic transmission that developed over a 20–25 min period, after which the suppression stabilized and was maintained for the remainder of the hypoxic epoch (Figure 1a). In a set of 12 paired experiments, there was no difference in the absolute degree of synaptic suppression between the two cohorts: synaptic transmission was suppressed by 73.81 ± 11.42% (mean ± SD) in WT slices, and 71.47 ± 21.31% in xCT^−/−^ slices (paired *t*_11 _= 0.541, *P* = 0.5991, Figure 1b). Ostensibly, these data suggest that hypoxia‐induced ATP breakdown and adenosine release are equivalent in WT and xCT^−/−^ mice.

### 3.2 xCT^−/−^ mice show accelerated rate of recovery and post‐hypoxic potentiation

Interestingly, although no difference in hypoxia‐induced neuronal suppression was observed between WT and xCT^−/−^ slices, mutant slices displayed faster recovery to 85% of baseline responses (*T*_85_) upon reoxygenation (paired *t*_11_*__* = 4.838, *P *= 0.0005, Figure 1c). Furthermore, while WT responses returned to 96.462 ± 10.9059% of pre‐hypoxic amplitudes, xCT^−/−^ slices showed post‐hypoxic potentiation reaching 135.898 ± 30.880% of pre‐hypoxic amplitudes: a significant increase when compared to WT slices (paired *t*_11 _= 3.587, *P* = 0.0043, Figure 1d). In fact, 11 out of 12 xCT^−/−^ slices reached at least 115% of their baseline amplitudes, whereas none of the WT slices exceeded baseline amplitudes by the same amount. This post‐hypoxic potentiation, hence referred to as ‘post‐hypoxic LTP’, peaked 30 min into reoxygenation and was maintained after 45 min of recovery. Both the accelerated rate of recovery and the post‐hypoxic LTP observed in xCT^−/−^ slices were unexpected findings, which required further investigation.

### 3.3 Post‐hypoxic LTP in xCT^−/−^ mice is not accompanied by changes in paired‐pulse facilitation

**FIGURE 2:**
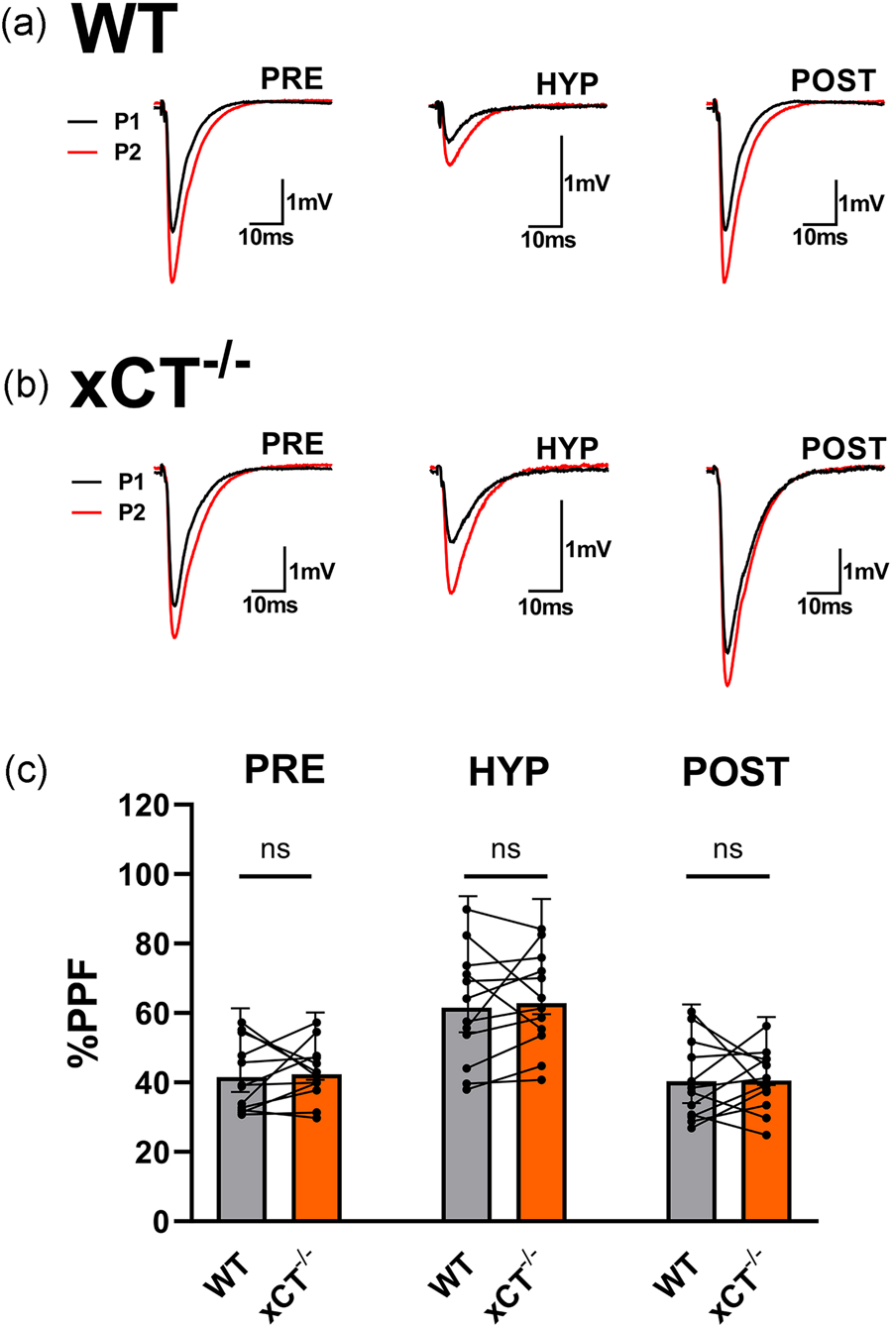
Post‐hypoxic LTP involves no change in paired‐pulse facilitation in xCT^−/−^ mice. (a, b) Representative traces (five averaged trials) for the first evoked response (P1; black) overlain with the facilitated response (P2; red) for WT (a) and xCT^−/−^ (b) slices during the pre‐hypoxic (PRE), hypoxic (HYP), and post‐hypoxic (POST) phases. Inter‐pulse interval (IPI) for the PPF stimulus was 75 ms. Note: vertical axis scale for HYP traces in (a, b) is altered to better highlight PPF). (c) Bar graph comparing the magnitude of PPF between WT and xCT^−/−^ slices for the pre‐hypoxic (PRE), hypoxic (HYP) and post‐hypoxic (POST) phases. PPF values are calculated as the percentage increase in the second pulse response, compared to the first. Data were generated from the paired experiments in Figure 1.

LTP induced by theta‐burst stimulation (TBS) is expressed by postsynaptic permutations in AMPA receptors (AMPARs) and does not alter paired‐pulse facilitation (PPF), a presynaptic form of short‐term plasticity (Muller & Lynch, 1989). In order to determine if post‐hypoxic potentiation in xCT^−/−^ mice is expressed in a similar manner as LTP, we measured the magnitude of PPF during the pre‐hypoxic (PRE), hypoxic (HYP) and post‐hypoxic (POST) phases of the experiments in Figure 1. First, we found no differences in PPF during the pre‐hypoxic (paired *t*_11_ = 0.456, *P* = 0.6571), hypoxic (paired *t*_11_ = 0.607, *P* = 0.5561), nor post‐hypoxic (paired *t*_11_ = 0.456, *P* = 0.6571) phases between WT and xCT^−/−^ slices (Figure 2c). Next, we performed a two‐way ANOVA with paired slices run as repeated measures for the genotype variable (WT vs. xCT^−/−^) and phase (PRE, HYP, POST) run as the within‐subjects variable. This yielded a significant main effect for phase (*F*_2,66_ = 25.79, *P* < 0.0001), but not for genotype (*F*_1,66_ = 0.192, *P* = 0.6628). The interaction effect between genotype and phase also failed to reach significance (*F*_2,66_ = 0.035, *P* = 0.9658). The hypoxic phase produced a significant increase in PPF compared to the pre‐ and post‐hypoxic phase for both WT (PRE: *P* = 0.0016, POST: *P* = 0.0006) and xCT^−/−^ mice (PRE: *P* = 0.0010, POST: *P* = 0.0002) (Šidák's test), consistent with a presynaptic mechanism of synaptic suppression during hypoxia (Coelho et al., 2000; Heit et al., 2021). Most importantly, however, xCT^−/−^ mice showed no change in PPF magnitude during post‐hypoxic potentiation when compared to pre‐hypoxic levels (*P* > 0.9999) (Šidák's test); ergo, this phenomenon and TBS‐induced LTP share a common expression mechanism.

### 3.4 Post‐hypoxic LTP and accelerated rate of recovery are NMDAR‐dependent in xCT^−/−^ mice

**FIGURE 3:**
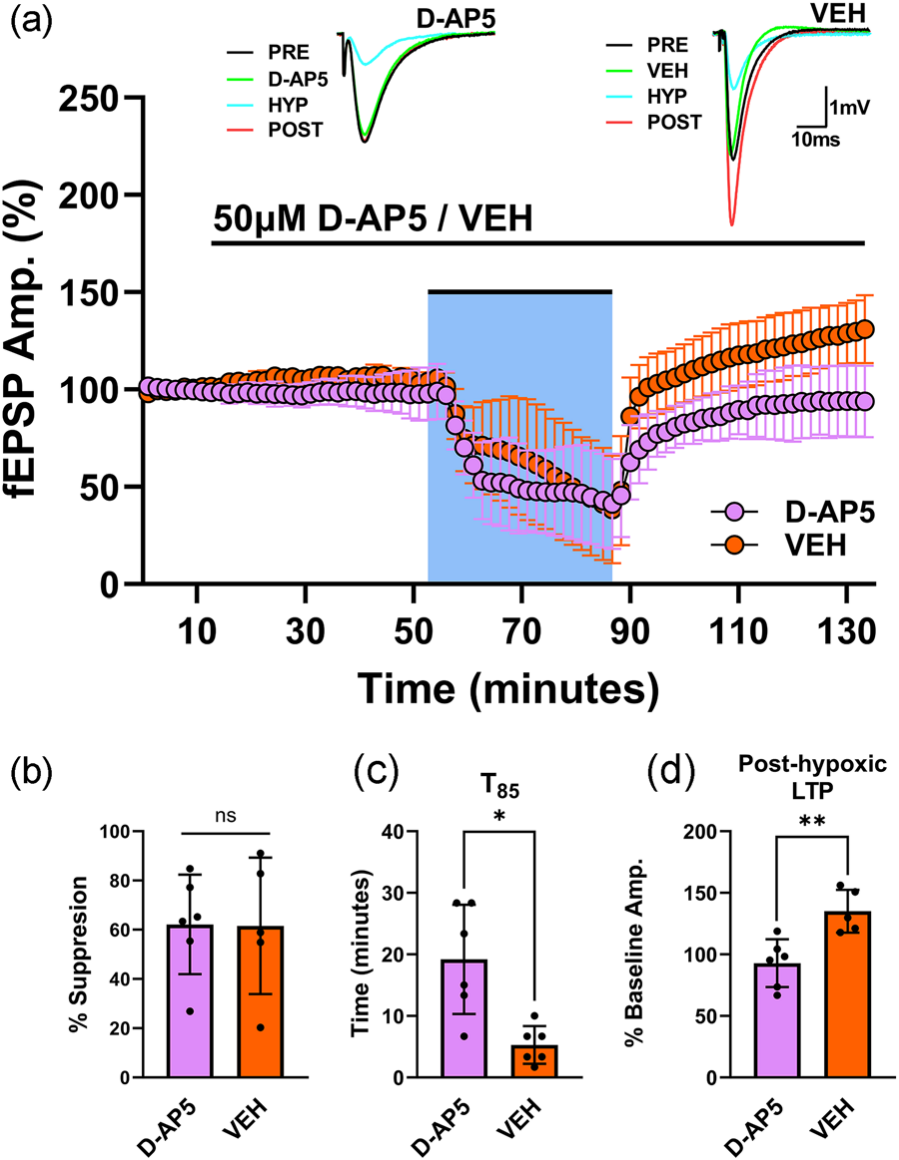
Post‐hypoxic LTP in xCT^−/−^ slices is prevented by antagonism of NMDA receptors. (a) Averaged data from hypoxia experiments, where xCT^−/−^ slices were pre‐treated and continuously perfused with 50 µM d‐AP5 (*n* = 6) or vehicle (VEH) (*n* = 5). Graph shows the effects of NMDAR antagonism on fEPSPs in xCT^−/−^ slices before, during (blue shaded area) and after 30 min of hypoxia (25% O_2_/75% N_2_). Each point is a mean of five consecutive trials for drug‐ (purple circles) or vehicle‐treated (orange circles) xCT^−/−^ slices within each experiment. Responses were normalized to the pre‐hypoxic baseline mean, and averaged (mean ± SD) across experiments at each point. Insets show averaged (*n* = 5) synaptic responses during the pre‐hypoxic baseline (PRE; immediately prior to hypoxia), treatment (d‐AP5 or VEH), hypoxic (HYP; last trials before re‐oxygenation), and post‐hypoxic (POST; 45 min after re‐oxygenation) phases for xCT^−/−^ slices in a representative experiment. Drug bar (d‐AP5/VEH) highlights the time course of d‐AP5 and vehicle treatments. (b) Bar graph (means ± SD) comparing the degree of fEPSP suppression for drug‐ and vehicle‐treated xCT^−/−^ slices during the hypoxic episode. (c) Bar graph (means ± SD) comparing the time required to attain 85% of baseline amplitude (*T*_85_) after re‐oxygenation in drug‐ and vehicle‐treated xCT^−/−^ slices. (d) Bar graph (means ± SD) comparing magnitude of post‐hypoxic LTP for drug‐ and vehicle‐treated xCT^−/−^ slices (data are expressed as percentage of baseline mean). 50 µM d‐AP5 effectively abolished post‐hypoxic LTP in xCT^−/−^ slices.

LTP induced by TBS requires the activation of postsynaptic voltage‐sensitive NMDARs (Collingridge et al., 1983) and calcium accumulation in the postsynaptic spine (Lynch et al., 1983; Malenka & Nicoll, 1993). The increase of calcium leads to a cascade of biochemical and molecular events, which perpetuate enhanced synaptic transmission (McNaughton, 1993). Importantly, the magnitude of this electrically induced form of potentiation resembles the magnitude of post‐hypoxic LTP observed in the xCT^−/−^ slices. In the hippocampal Schaffer collateral pathway, LTP is induced by NMDAR activation and can be abolished by the NMDAR antagonist, d‐2‐amino‐5‐phosphonopentanoic acid (d‐AP5) (Collingridge et al., 1983). We therefore tested whether this NMDAR‐dependent mechanism was taking place in xCT^−/−^ slices as a result of hypoxia. After establishing baseline recordings, xCT^−/−^ slices were perfused with either 50 µM d‐AP5 or vehicle, subjected to 30 min of hypoxia, fully reoxygenated, and allowed to recover for 45 min (Figure 3). During the hypoxic episode, synaptic transmission was suppressed by 62.149% ± 20.194 in drug‐treated slices and 61.582% ± 21.723 in vehicle‐treated slices: there were no differences in synaptic suppression between the two conditions (*t*_9 _= 0.039, *P* = 0.9695, Figure 3b). Upon reoxygenation, however, xCT^−/−^ slices treated with d‐AP5 returned to 92.890% ± 19.395 of pre‐hypoxic amplitudes, while vehicle‐treated slices reached 135.040% ± 17.391 of pre‐hypoxic amplitudes. Administration of 50 µM d‐AP5 in xCT^−/−^ slices effectively blunted the rate of recovery (*t*_9 _= 3.627 *p* = 0.0046, Figure 3c) and abolished post‐hypoxic LTP (*t*_9 _= 3.764, *P* = 0.0045, Figure 3d) when compared to vehicle‐treated slices.

### 3.5 WT and xCT^−/−^ mice do not differ in TBS‐induced LTP

**FIGURE 4:**
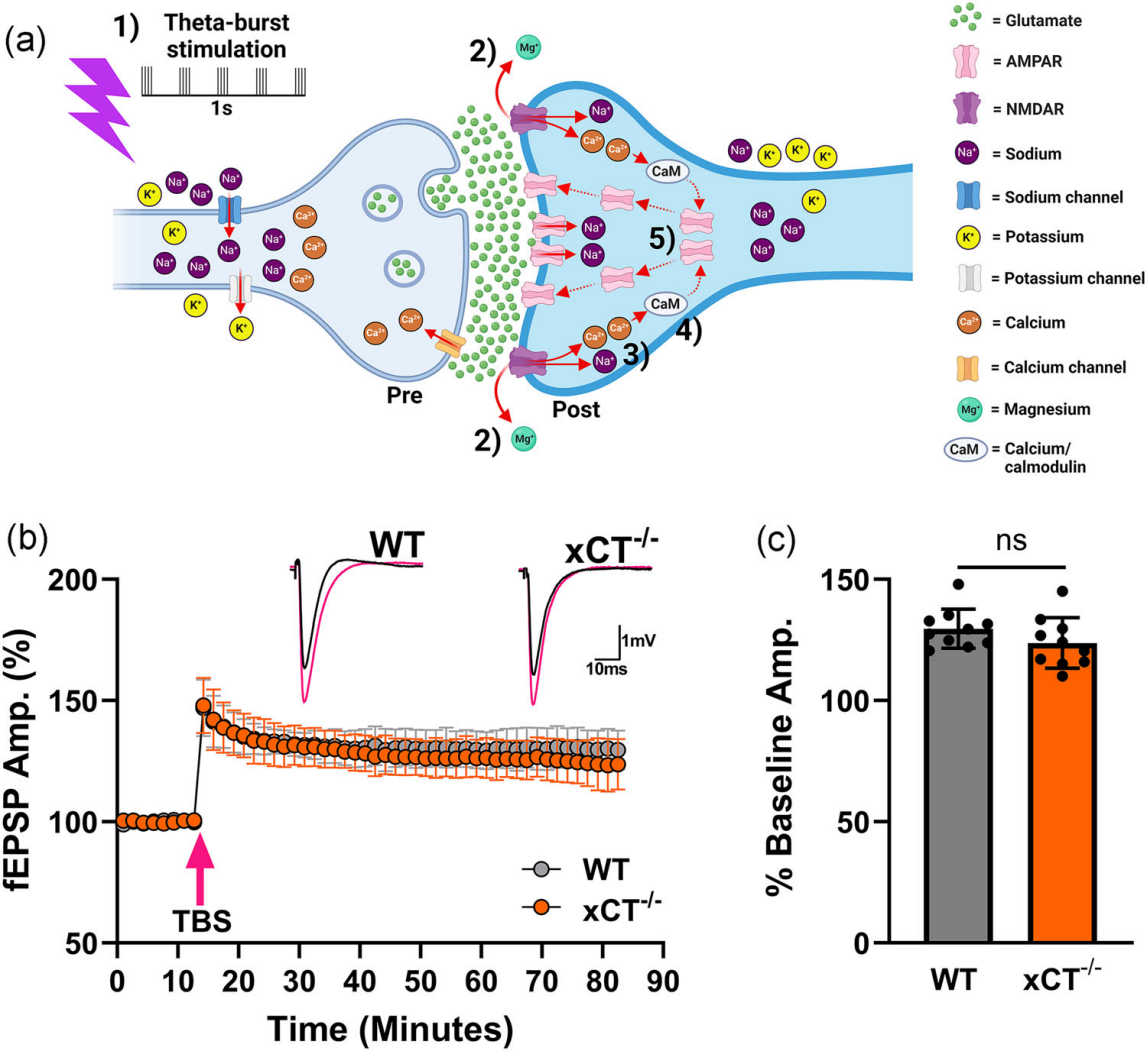
WT and xCT^−/−^ mice do not differ in theta‐burst stimulated LTP. (a) Schematic illustration of the proposed mechanism for theta‐burst stimulation (TBS)‐induced LTP. TBS is given as two episodes of five theta‐bursts (four pulses at 100 Hz with 200 ms between the bursts) separated by 5 s. (1) TBS triggers a surge of presynaptic glutamate release which (2) depolarizes postsynaptic neurons enough to remove the magnesium block from voltage‐sensitive NMDARs. (3) This allows sodium and calcium to penetrate the postsynaptic neuron. (4) Intracellular calcium then activates calcium/calmodulin (CaM) which triggers numerous molecular cascades leading to (5) the increased trafficking and insertion of AMPARs into the postsynaptic membrane thus enhancing fEPSPs. (b) Averaged data from 10 paired experiments where WT and xCT^−/−^ slices were subjected to TBS. Graph shows the effects of TBS (magenta arrow) on synaptic transmission for WT (grey) and xCT^−/−^ (orange) slices. Each point is a mean of five consecutive trials for WT and xCT^−/−^ slices. Responses were normalized to the baseline mean, and averaged (mean ± SD) across experiments at each point. Insets show averaged (*n* = 5) synaptic responses at the baseline (black) and 60 min post‐TBS (magenta). (c) Bar graph (means ± SD) comparing magnitude of TBS‐induced LTP for WT and xCT^−/−^ slices (data are expressed as percentage of pre‐TBS baseline). No difference was observed between genotypes.

Because the post‐hypoxic LTP observed in mutant slices was NMDAR‐dependent, we next investigated whether WT and xCT^−/−^ mice differ in their response to TBS‐induced LTP. After establishing baseline responses, two episodes of five theta‐bursts (four pulses at 100 Hz with 200 ms between the bursts) separated by 5s were given to area CA1 in both WT and xCT^−/−^ slices (Figure 4b). Recording was continued for 60 min after TBS stimulus to monitor the LTP induced. The mean field excitatory postsynaptic potential (fEPSP) amplitude after TBS intervention increased to 129.594 ± 8.037% of baseline in WT slices and 123.747 ± 10.400% of baseline in xCT^−/−^ slices (Figure 4c). There was no difference in the magnitude of LTP induced between genotypes 60 min after TBS intervention (paired *t*_18 _= 1.407, *P* = 0.1765, Figure 4c). These data are in contrast to one prior report which found attenuated LTP in xCT‐deficient mice (Li et al., 2012). This study, however, employed high frequency stimulation to induce LTP, used the *sut/sut* mouse model, and incubated slices in a submerged chamber, which may account for the discrepancy.

### 3.6 Post‐hypoxic LTP in xCT^−/−^ slices is unaffected by A_1_R antagonism

**FIGURE 5:**
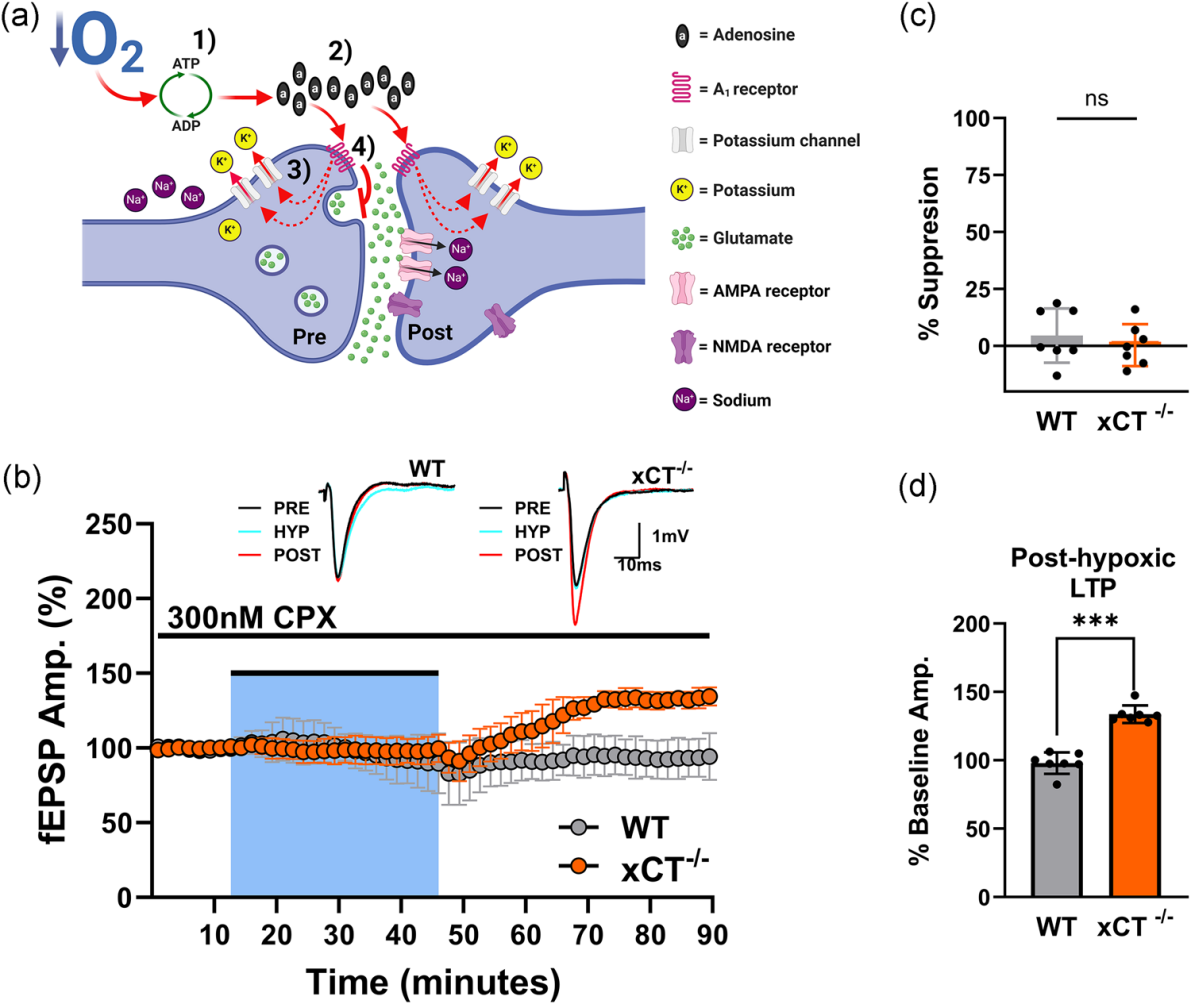
Post‐hypoxic LTP in xCT^−/−^ mice is unaffected by A_1_R antagonism. (a) Schematic illustration of the effects of hypoxia‐induced ATP breakdown and adenosine release. (1) Hypoxia causes breakdown of ATP to ADP and adenosine. (2) Expelled adenosine activates A_1_Rs, (3) which opens K^+^ channels, thus hyperpolarizing the terminal and (4) inhibiting presynaptic glutamate release. (b) Averaged data from seven paired experiments show the effects of hypoxia on fEPSPs in WT and xCT^−/−^ slices pretreated with 300 nM CPX. Each point is a mean of five consecutive trials for WT (grey) and xCT^−/−^ (orange) slices within each experiment, normalized to the pre‐hypoxic baseline mean, and averaged (mean ± SD) across experiments at each point. Hypoxia (25% O_2_/75% N_2_) was applied for 30 min (blue shaded area). Insets show averaged (*n* = 5) synaptic responses during the pre‐hypoxic baseline (PRE; immediately prior to hypoxia), hypoxic (HYP; last trials before re‐oxygenation) and post‐hypoxic (POST; 30 min after re‐oxygenation) phases for WT and xCT^−/−^ slices. CPX administration was continuous throughout the hypoxic and post‐hypoxic phase. (c) Bar graph (means ± SD) comparing percentage of hypoxia‐induced synaptic suppression for WT and xCT^−/−^ slices with CPX treatment. Data represent the magnitude of responses measured just prior to reoxygenation. (d) Bar graph (means ± SD) comparing magnitude of post‐hypoxic LTP for WT and xCT^−/−^ slices after CPX treatment (data are expressed as percentage of baseline mean).

During hypoxia, the release of adenosine due to ATP breakdown inhibits presynaptic glutamate release via the activation of presynaptic A_1_Rs (Figure 5a), which suppresses synaptic transmission. Although the degree of synaptic suppression during hypoxia did not differ between WT and xCT^−/−^ slices, the downstream consequences of said suppression, and/or the neuromodulatory effects of adenosine, may differ between genotypes. In normoxia, the presence of endogenous adenosine has been shown to dampen (Arai et al., 1990; Forghani & Krnjevic, 1995) as well as enhance (Fuji, 2004; Fuji et al., 2002; Yamazaki & Fuji, 2015) NMDAR‐dependent LTP in the hippocampus. The potential role of *hypoxia‐induced* adenosine release in the generation of LTP, however, has yet to be scrutinized. As such, we tested whether the inhibition of hypoxia‐induced synaptic suppression via adenosine antagonism would affect post‐hypoxic LTP in xCT^−/−^ mice.

Paired experiments were performed, where WT and xCT^−/−^ slices were pretreated with the potent and selective adenosine A_1_R antagonist 8‐cyclopentyl‐1,3‐dipropylxanthine (CPX) for 40 min. After ≥12 min of baseline recordings, slices were subjected to 30 min hypoxia, and allowed to recover for 45 min. CPX was dosed at 300 nM as this is sufficient to retain complete preservation of the synaptic response during hypoxia (Heit et al., 2021). Drug administration was continuous throughout the hypoxic and recovery phase. CPX effectively abolished hypoxia‐induced synaptic suppression in both genotypes for the duration of hypoxia (Figure 5b), and, accordingly, no difference in synaptic suppression was observed between cohorts (paired *t*_6_ = 1.124, *P* = 0.3040, Figure 5c). Upon reoxygenation, xCT^−/−^ slices once again displayed post‐hypoxic LTP when compared to WT slices (paired *t*_6_ = 11.470, *P *< 0.0001, Figure 5d). Mutant slices potentiated to 133.672 ± 6.409% of baseline responses, while WT slices reached 97.937 ± 7.823% and were hence unchanged from baseline levels.

### 3.7 Pharmacological antagonism of system x_c_^−^ in WT slices reproduces post‐hypoxic LTP observed in xCT KO slices

**FIGURE 6:**
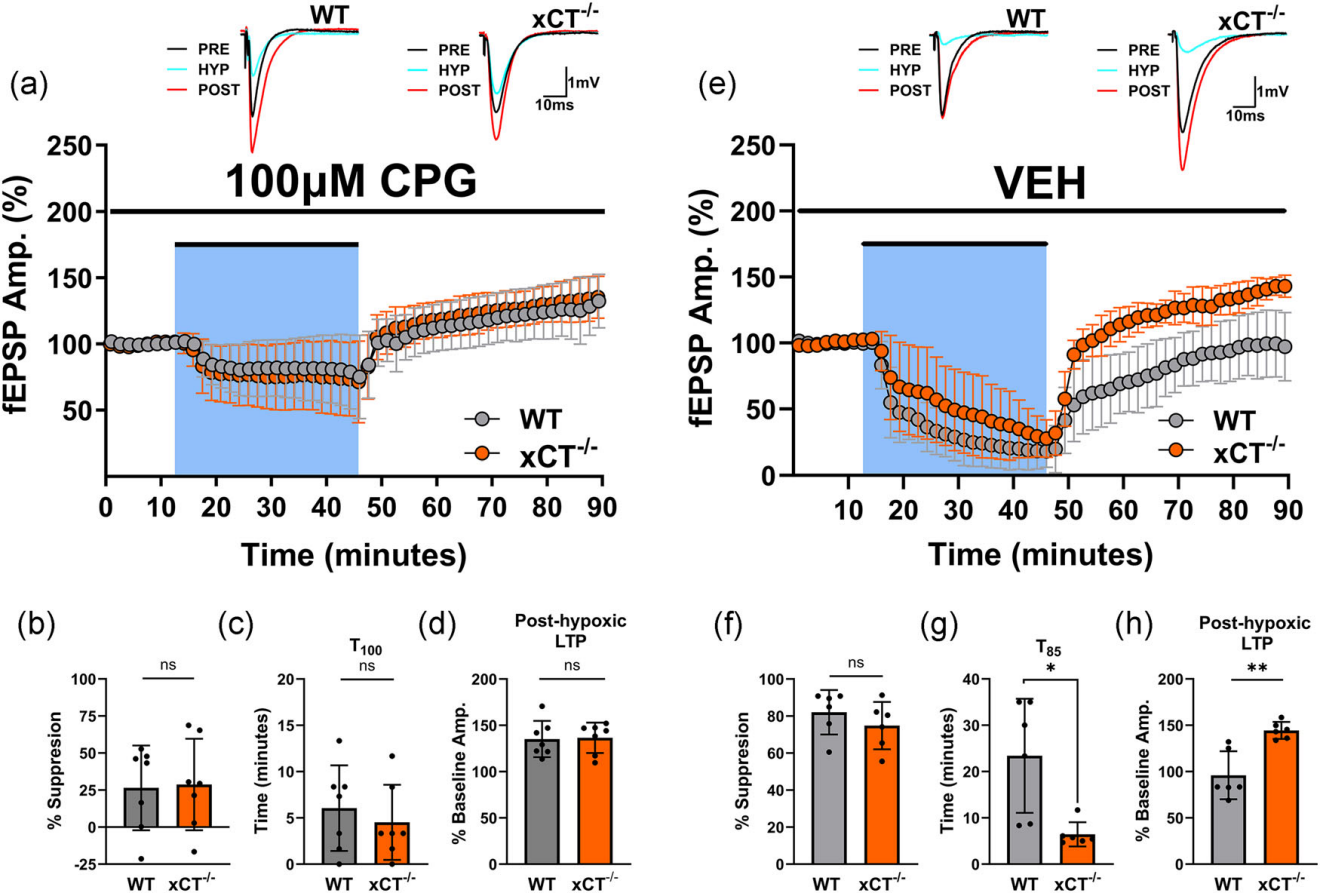
Pharmacological antagonism of system x*_c_* ^−^ in WT mice reproduces post‐hypoxic LTP observed in xCT^−/−^ mice. Left side: (a) Averaged data from seven paired experiments showing the effects of hypoxia on fEPSPs in WT and xCT^−/−^ slices after 2 h pretreatment with 100 µM CPG. Each point is a mean of five consecutive trials for WT (grey) and xCT^−/−^ (orange) slices within each experiment, normalized to the pre‐hypoxic baseline mean, and averaged (mean ± SD) across experiments at each point. Hypoxia (25% O_2_/75% N_2_) was applied for 30 min (blue shaded area). Insets show averaged (*n* = 5) synaptic responses during the pre‐hypoxic baseline (PRE; immediately prior to hypoxia), hypoxic (HYP; last trials before re‐oxygenation) and post‐hypoxic (POST; 45 min after re‐oxygenation) phases for WT and xCT^−/−^ slices in a representative experiment. CPG administration was continuous throughout the hypoxic and post‐hypoxic phase. (b) Bar graph (means ± SD) comparing the degree of fEPSP suppression for WT and xCT^−/−^ slices during the hypoxic episode. (c) Bar graph (means ± SD) comparing the time required to attain 100% of pre‐hypoxic baseline amplitude (*T*_100_) after re‐oxygenation for WT and xCT^−/−^ slices. (d) Bar graph (means ± SD) comparing magnitude of post‐hypoxic LTP for WT and xCT^−/−^ slices after CPG treatment. Right side: (e) Averaged data from six paired experiments showing the effects of hypoxia on fEPSPs in WT and xCT^−/−^ slices after 2 h pretreatment with CPG vehicle 1.0 M NaOH (VEH). Each point is a mean of five consecutive trials for WT (grey) and xCT^−/−^ (orange) slices within each experiment, normalized to the pre‐hypoxic baseline mean, and averaged (mean ± SD) across experiments at each point. Hypoxia (25% O_2_/75% N_2_) was applied for 30 min (blue shaded area). Insets show averaged (*n* = 5) synaptic responses during the pre‐hypoxic baseline (PRE; immediately prior to hypoxia), hypoxic (HYP; last trials before re‐oxygenation) and post‐hypoxic (POST; 45 min after re‐oxygenation) phases for WT and xCT^−/−^ slices. Vehicle administration was continuous throughout the hypoxic and post‐hypoxic phase. (f) Bar graph (means ± SD) comparing the degree of fEPSP suppression for WT and xCT^−/−^ slices during the hypoxic episode. (g) Bar graph (means ± SD) comparing the time required to attain 85% of pre‐hypoxic baseline amplitude (*T*_85_) after re‐oxygenation for WT and xCT^−/−^ slices. (h) Bar graph (means ± SD) comparing magnitude of post‐hypoxic LTP for WT and xCT^−/−^ slices after vehicle treatment.

Since the genetic deletion of system x_c_^−^ promotes the generation of post‐hypoxic LTP, we aimed to determine whether pharmacological antagonism of system x_c_^−^ would cause WT slices to mimic the potentiation observed in xCT^−/−^ slices. We therefore performed paired experiments where WT and xCT^−/−^ slices were pretreated with *S*‐4‐carboxyphenylglycine (CPG, 100 µM), a non‐substrate inhibitor of the antiporter (Bridges et al., 2012; Patel et al., 2004), for 2 h prior to the 30 min hypoxic episode, and then measured recovery 45 min after reoxygenation. Drug administration was continuous throughout the hypoxic and post‐hypoxic phase. The 2 h pretreatment was selected in light of prior investigations suggesting this to be the required epoch to effectively inhibit the antiporter's principal downstream effect of supplying ambient glutamate (Heit et al., 2022). CPG treatment caused WT slices to phenocopy the rate of recovery (paired *t*_6 _= 0.0266, *P* = 0.9797, Figure 6c) and the post‐hypoxic LTP (paired *t*_6 _= 0.115, *P* = 0.9125, Figure 6d) observed in xCT^−/−^ slices, thus eliminating the difference between genotypes. (Here, rate of recovery was measured as the time required to attain 100% of baseline responses after reoxygenation, as suppression did not reach 15% in several experiments during hypoxic episode.) Specifically, WT slices potentiated to 135.142 ± 19.556%, and xCT^−/−^ slices potentiated to 136.512 ± 16.483% upon re‐oxygenation. Although hypoxia‐induced synaptic suppression did not differ between WT and xCT^−/−^ mice (paired *t*_6 _= 0.241, *P* = 0.8179, Figure 6b), CPG administration attenuated the magnitude of suppression in both genotypes (see below). When experiments were performed with vehicle, responses reproduced control conditions: xCT^−/−^ slices showed accelerated rate of recovery (paired *t*_5 _= 3.704, *P* = 0.0139, Figure 6g) and post‐hypoxic LTP (paired *t*_5 _= 4.642, *P* = 0.00056, Figure 6h) when compared to WT slices, which did not exceed baseline responses. Specifically, xCT^−/−^ slices potentiated to 144 ± 9.104% of baseline responses, while WT slices reached 95.999 ± 25.9266%, thus remaining at baseline levels (Figure 6e). As expected, there was no difference in hypoxia‐induced synaptic suppression between genotypes (paired *t*_5 _= 1.957, *P *= 0.1077, Figure 6f) during vehicle conditions.

A two‐way ANOVA was performed with paired slices run as repeated measures for the genotype variable (WT vs. xCT^−/−^) and drug treatment (VEH vs. CPG) run as the between‐subjects variable. This yielded a significant main effect for both genotype (*F*_1,22 _= 24.53, *P *= 0.0055) and for drug treatment (*F*_1,22_ = 9.676, *P* = 0.0452) on post‐hypoxic LTP. The interaction effect between genotype and drug treatment was also significant (*F*_1,22 _= 21.90, *P* = 0.042). CPG had a significant effect in enabling post‐hypoxic LTP in WT slices (*P *= 0.0065), but not xCT^−/−^ slices (*P* = 0.9743) (Šidák's test). The lack of drug effect in xCT^−/−^ slices strongly supports the conclusion that its action in WT slices was due to inhibition of system x_c_^−^ and not a non‐specific drug effect. An additional two‐way ANOVA was performed to assess the drug's action on synaptic suppression during the hypoxic insult. This yielded a significant main effect for drug treatment (*F*_1,22 _= 56.160, *P *< 0.0001), but not for genotype (*F*_1,22_ = 0.0641, *P* = 0.8579). The interaction between genotype and drug treatment (VEH vs. CPG) also failed to reach significance (*F*_1,22 _= 0.636, *P* = 0.5750). Interestingly, CPG significantly inhibited hypoxia‐induced synaptic suppression in both WT slices (*P *= 0.0023) and xCT^−/−^ slices (*P* = 0.0161) (Šidák's test).

### 3.8 Paired‐pulse facilitation is unaltered during CPG‐induced post‐hypoxic LTP

**FIGURE 7:**
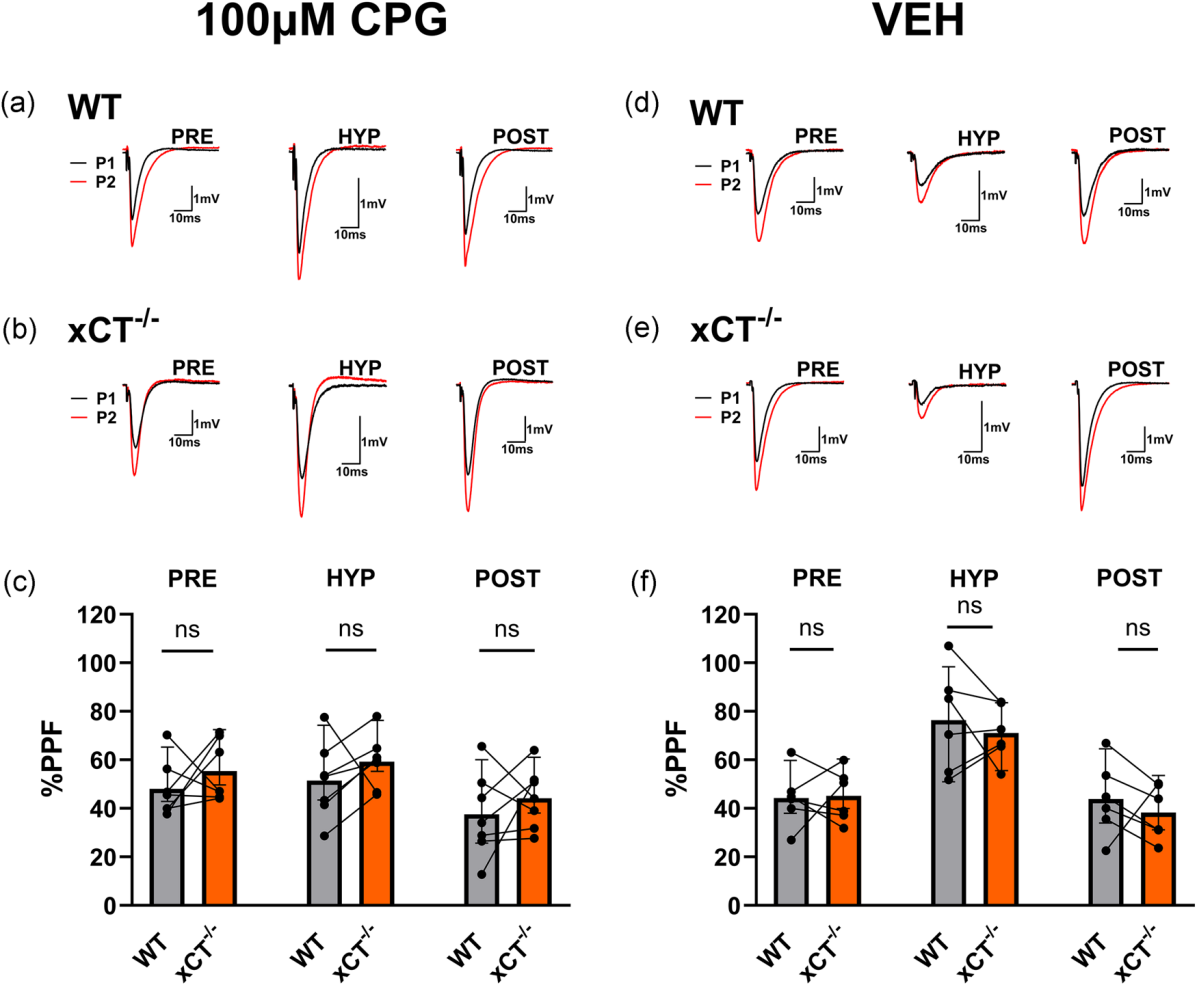
CPG‐induced post‐hypoxic LTP in WT mice involves no change in paired‐pulse facilitation. Left side: (a, b) Representative traces (five averaged trials) for the first evoked response (P1; black) overlain with the facilitated response (P2; red) for WT (a) and xCT^−/−^ (b) slices during the pre‐hypoxic (PRE), hypoxic (HYP) and post‐hypoxic (POST) phases of CPG treatment. Inter‐pulse interval (IPI) for the PPF stimulus was 75 ms. Note: vertical axis scale for HYP traces in (a, b) is altered for consistency). (c) Bar graph (means ± SD) comparing the magnitude of PPF between WT and xCT^−/−^ slices for the pre‐hypoxic (PRE), hypoxic (HYP) and post‐hypoxic (POST) phases. PPF values are calculated as the percentage increase in the second pulse response, compared to the first. Right side: (d, e) Representative traces (five averaged trials) for the first evoked response (P1; black) overlain with the facilitated response (P2; red) for WT (d) and xCT^−/−^ (e) slices during the pre‐hypoxic (PRE), hypoxic (HYP) and post‐hypoxic (POST) phases of vehicle (VEH) treatment. Inter‐pulse interval (IPI) for the PPF stimulus was 75 ms. Note: vertical axis scale for HYP traces in (d, e) is altered for consistency). (f) Bar graph (means ± SD) comparing the magnitude of PPF between WT and xCT^−/−^ slices for the pre‐hypoxic (PRE), hypoxic (HYP) and post‐hypoxic (POST) phases. PPF values are calculated as the percentage increase in the second pulse response, compared to the first. Data were generated from the paired experiments in Figure 6.

We also measured the magnitude of PPF during the pre‐hypoxic (PRE), hypoxic (HYP) and post‐hypoxic (POST) phases with CPG treatment. First, as shown in Figure 7a−c, we found that WT and xCT^−/−^ slices did not differ in PPF for the pre‐hypoxic (paired *t*_6_ = 0.949, *P* = 0.3794), hypoxic (paired *t*_6_ = 1.156, *P* = 0.2914), nor post‐hypoxic (paired *t*_6_ = 0.866, *P* = 0.4204) phases. Next, we performed a two‐way ANOVA with paired slices run as repeated measures for the genotype variable (WT vs. xCT^−/−^) and phase (PRE, HYP, POST) run as the within‐subjects variable. This yielded a significant main effect for phase (*F*_2,36_ = 4.279, *P* = 0.0215), but not for genotype (*F*_1,36_ = 2.931, *P* = 0.0955). The interaction effect between genotype and phase also failed to reach significance (*F*_2,36_ = 0.006, *P* = 0.9944). Interestingly, under CPG treatment, the hypoxic phase did not produce an increase in PPF compared to the pre‐ and post‐hypoxic phase for either WT (PRE: *P* > 0.9999, POST: *P* = 0.6827) or xCT^−/−^ mice (PRE: *P* > 0.9999, POST: *P* = 0.5950) (Šidák's test). This is undoubtedly due to the attenuated hypoxia‐induced suppression of synaptic transmission by CPG in both genotypes. Since this attenuation occurred in xCT^−/−^ slices, it likely represents an off‐target consequence of the system x_c_^−^ inhibitor. Most importantly, however, neither WT (*P* = 0.9700) nor xCT^−/−^ (*P* = 0.8995) mice showed an alteration in PPF magnitude during CPG‐induced post‐hypoxic LTP when compared to pre‐hypoxic levels (Šidák's test).

In vehicle conditions, the PPF responses returned to those of control experiments (Figures 1 and 2). As shown in Figure 7d–f, there were no differences in PPF between WT and xCT^−/−^ slices during the pre‐hypoxic (paired *t*_5_ = 0.150, *P* = 0.8867), hypoxic (paired *t*_5_ = 0.703, *P* = 0.5133), nor post‐hypoxic (paired *t*_5_ = 0.823, *P *= 0.4478) phases. A two‐way ANOVA was performed with paired slices run as repeated measures for the genotype variable (WT vs. xCT^−/−^) and phase (PRE, HYP, POST) run as the within‐subjects variable. This yielded a significant main effect for phase (*F*_2,30_ = 19.250, *P* < 0.0001), but not for genotype (*F*_1,30_ = 0.502, *P* = 0.4840). The interaction effect between genotype and phase also failed to reach significance (*F*_2,30_ = 0.201, *P* = 0.8191). Under vehicle treatment, the hypoxic phase produced an increase in PPF compared to the pre‐ and post‐hypoxic phase for both WT (PRE: *P* > 0.0067, POST: *P* = 0.0060) and xCT^−/−^ mice (PRE: *P* > 0.0458, POST: *P* = 0.0055) (Šidák's test). Once again, as in control conditions, xCT^−/−^ mice showed no alteration in PPF magnitude during post‐hypoxic LTP when compared to pre‐hypoxic levels (*P* > 0.9999) (Šidák's test).

### 3.9 Post‐hypoxic LTP can be induced in WT slices incubated with high Ca^2+^ ACSF

Post‐hypoxic LTP was only observed in slices from mice lacking xCT or in WT slices where system x_c_^−^ was inhibited by CPG. Prior studies, however, have demonstrated LTP‐like effects in non‐mutant animals after episodes of severe oxygen deprivation (anoxia) that triggered anoxic depolarization (Crépel et al., 1993; Crepel et al., 1993; Arcangeli et al., 2013; Gozlan et al., 1994; Hsu & Huang, 1997; Maggio et al., 2015). Because post‐synaptic calcium influx is critical for induction of NMDAR‐dependent LTP, we reasoned that post‐hypoxic LTP might be enabled in WT slices incubated in elevated [Ca^2+^]. To test this, WT slices were bathed in ACSF medium containing 1, 2, or 4 mM calcium (with [Mg^2+^] held constant at 2 mM), subjected to 30 min of hypoxia (25% O_2_/75% N_2_), re‐oxygenated and allowed to recover for 30 min. Altogether, calcium concentration showed a dose‐dependent effect on the magnitude of synaptic suppression, rate of recovery and post‐hypoxic potentiation in WT slices.

**FIGURE 8:**
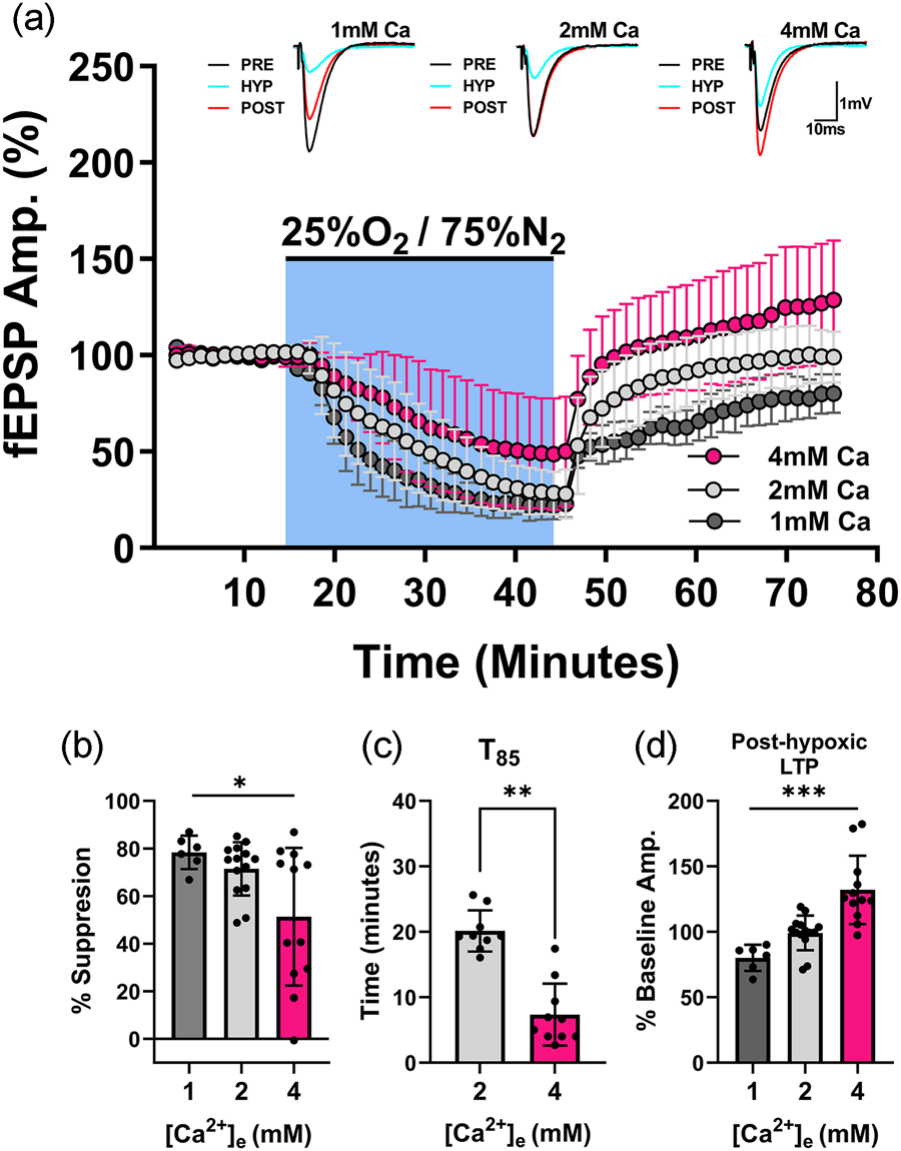
Post‐hypoxic LTP in WT mice is Ca^2+^ dependent. (a) Averaged data from hypoxia experiments, where WT slices were bathed in ACSF containing calcium concentrations of 1 mM (*n* = 6), 2 mM (*n* = 14) or 4 mM (*n* = 12). Concentrations of all other elements remained constant. Graph shows the effect of calcium concentration before, during (blue shaded area) and after 30 min of hypoxia (25% O_2_/75% N_2_). Each point is a mean of four consecutive trials for experiments using 1 mM (dark grey circles), 2 mM (light grey circles) or 4 mM (magenta circles) calcium ACSF. Responses were normalized to the pre‐hypoxic baseline mean, and averaged (mean ± SD) across experiments at each point. Insets show averaged (*n* = 4) synaptic responses during the pre‐hypoxic baseline (PRE; immediately prior to hypoxia), hypoxic (HYP; last trials before re‐oxygenation) and post‐hypoxic (POST; 30 min after re‐oxygenation) phases. (b) Bar graph (means ± SD) comparing the degree of fEPSP suppression for all three conditions during the hypoxic episode. (c) Bar graph (means ± SD) comparing the time required to attain 85% of baseline amplitudes (*T*_85_) after re‐oxygenation in WT slices bathed in 2 and 4 mM calcium. Slices bathed in 4 mM calcium ACSF showed an accelerated rate of recovery when comparing to WT slices bathed in 2 mM ACSF. (Data from slices bathed in 1 mM calcium were omitted for this comparison as they failed to reach 85% of baseline amplitudes.) (d) Bar graph (means ± SD) comparing magnitude of post‐hypoxic LTP for all three ACSF conditions. Extracellular calcium concentration significantly affected post‐hypoxic LTP such that higher levels of calcium produced enhanced potentiation.

Figure 8 summarizes the results from a total of 32 hypoxia experiments with WT slices in these three conditions. Firstly, the level of calcium concentration had a significant effect on the magnitude of synaptic suppression during hypoxia (*F*_2,29 _= 12.691, *P* < 0.0190, ANOVA, Figure 8b). Responses from WT slices bathed in the 1 mM calcium ACSF failed to reach 100% recovery after re‐oxygenation and displayed post‐hypoxic depression (Figure 8a, b), whereas responses from WT slices bathed in 2 mM calcium ACSF displayed a ‘normal’ rate of recovery and returned to pre‐hypoxic baseline amplitudes (Figure 8a,c,d), thus matching the responses of WT slices from paired experiments with xCT^−/−^ slices (Figure 1), where the extracellular calcium concentration was also 2 mM (Figure 1a,c,d). Interestingly, responses from WT slices bathed in 4 mM calcium ACSF showed an accelerated rate of recovery when compared to WT slices bathed in 2 mM ACSF (*t*_24 _= 3.934, *P* = 0.0008, Figure 8c). Most notably, however, statistical analyses revealed a highly significant effect of extracellular calcium concentration on post‐hypoxic LTP such that higher levels of calcium produced enhanced potentiation (*F*_2,29 _= 18.023, *P *< 0.0001, ANOVA, Figure 8a,d). Further comparisons showed a significant increase in post‐hypoxic LTP in WT slices bathed in 4 mM calcium ACSF when compared to those bathed in 1 mM (*P *< 0.0001, Figure 8d) and 2 mM (*P* = 0.0003, Figure 8d) calcium ACSF (Tukey's multiple comparisons). In sum, WT slices display both accelerated rate of recovery and post‐hypoxic LTP when bathed in 4 mM calcium; therefore, responses from WT slices bathed in 4 mM (‘high’) calcium ACSF (Figure 8a,c,d) reproduced responses from xCT^−/−^ slices bathed in 2 mM calcium ACSF (Figure 1a,c,d).

**FIGURE 9:**
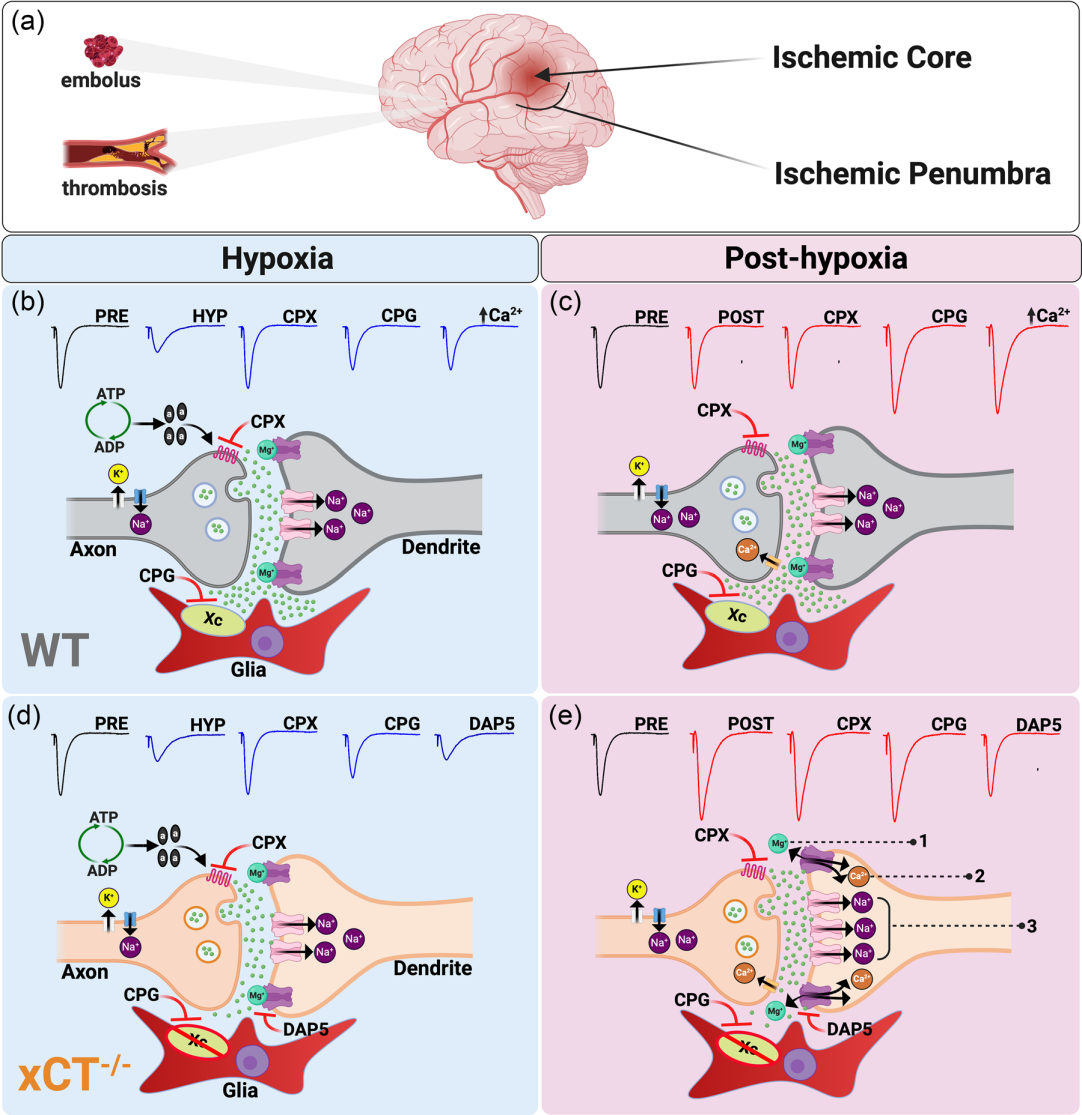
(a) When a cerebral blood vessel becomes occluded during stroke, two distinct regions form: the ischemic *core* and the ischemic *penumbra*. The core suffers severe oxygen deprivation (anoxia), glutamate excitotoxicity, anoxic depolarization, and rapid cell death. The penumbra, which is the outer rim of the core, experiences mild ischemia (hypoxia) and altered synaptic signaling but retains the potential to be spared via adaptations in neuronal excitability. (b‐e) Employing hippocampal slice electrophysiology, we modelled the ischemic penumbra by subjecting wild‐type (WT) mice and system x_c_^−^ knockout (xCT^−/−^) mice to hypoxia. When compared to pre‐hypoxia baseline responses (PRE), adenosine‐mediated suppression of synaptic responses during hypoxia (HYP) was equivalent between genotypes. xCT^−/−^ responses, however, displayed potentiation in the post‐hypoxia phase (POST) which resembled LTP. This post‐hypoxic LTP was abolished by NMDAR antagonist DAP5, but unaffected by A_1_R antagonist CPX and system x_c_^−^ inhibitor CPG. WT responses phenocopied post‐hypoxic LTP after treatment with CPG or when incubated in elevated Ca^2+^ concentrations. Collectively, these data suggest that post‐hypoxic LTP with system x_c_^−^ interference is driven by enhanced postsynaptic depolarization and 1) removal of Mg^2+^ from NMDARs which 2) allows Ca^2+^ influx thus 3) increasing AMPAR insertion.

## 4 DISCUSSION

Neuropathological investigations have established that strokes involving spatially restricted cerebral ischaemia produce localized patterns of cellular reactions that differ in the ischaemic core versus the ischaemic penumbra. The severe oxygen deprivation within the ischaemic core precipitates an excitotoxic cascade and rapid cell death (Lipton, 1999), whereas hypoxic penumbral tissue, the hypo‐perfused outer rim of the core (Ramos‐Cabrer et al., 2011), can exhibit adaptations in neuronal excitability which spark recovery. There are currently no therapies available to mitigate damage within the ischaemic core; however, penumbral tissue retains the potential to be spared via the induction of neuroplastic mechanisms (Li et al., 2020; Liu et al., 2019; Shen et al., 2021; Wang et al., 2021).

In our previous report (Heit et al., 2022), which used total oxygen deprivation (anoxia) to simulate stroke in hippocampal slices, we found that tonic, system x_c_^−^ secreted glutamate drives both the rapidity and synchrony of depolarizing events after anoxia. Both genetic deletion and pharmacological antagonism of system x_c_^−^ provided ischaemic neuroprotection by increasing latency to anoxic depolarization (AD), attenuating AD wave amplitudes, and extending AD wave durations (Heit et al., 2022). Our data supported the conclusion that the antiporter's obligate release of ambient glutamate exacerbates the ischaemic core. Importantly, recent studies have also highlighted the potential role of system x_c_^−^ in the ischaemic penumbra. Both middle cerebral artery occlusion (MCAO) (Domercq et al., 2016; Hsieh et al., 2017; Soria et al., 2014) and photothrombotic (He & Hewitt, 2022) stroke models reveal that system x_c_^−^ becomes robustly upregulated in the days after ischaemic insult; and antagonism of the antiporter significantly mitigates infarct volume (He & Hewitt, 2022) and improves post‐stroke functional recovery (Domercq et al., 2016; Hsieh et al., 2017). Considering these findings, we hypothesized that system x_c_^−^ inhibition may also trigger neuroplastic mechanisms which foster recovery in the penumbra.

The current study therefore scrutinized system x_c_^−^ function during and after hypoxia as a means to explicate the antiporter's potential role in penumbral tissue. Hippocampal slices prepared from WT mice and transgenic mice lacking a functional system x_c_^−^ (xCT^−/−^ mice) were challenged with an episode of mild hypoxia and allowed to recover. Surprisingly, although the steady‐state depression of synaptic transmission by mild hypoxia did not differ between WT and xCT^−/−^ slices, we observed post‐hypoxic potentiation specific to xCT^−/−^ mice. This was a striking result as theta‐burst stimulation (TBS)‐induced LTP was equivalent between genotypes. A deeper probe into the induction and expression mechanisms of this hypoxia‐mediated plasticity revealed that post‐hypoxic potentiation in xCT^−/−^ slices was not driven by an adenosine‐sensitive pathway, but rather the activation of NMDARs, as well as increased calcium influx, with no change in paired‐pulse facilitation (PPF). Hence, the observed phenomenon engaged similar mechanisms as LTP evoked by high frequency synaptic stimulation (e.g. TBS). Importantly, the pharmacological inhibition of system x_c_^−^ in WT slices reproduced the post‐hypoxic effect observed in mutants. Collectively, these data yield salient implications for post‐stroke rehabilitation in the penumbral zone and may reveal an upstream mechanism which drives the enhanced functional recovery in rodents where system x_c_^−^ is pharmacologically inhibited (Domercq et al., 2016; Hsieh et al., 2017).

### 4.1 The xCT^−/−^ mouse model

The glial‐bound (Ottestad‐Hansen et al., 2018) cystine/glutamate antiporter, system x_c_^−^, supplies the majority of extracellular glutamate in the brain (Baker et al., 2002; DeBundel et al., 2011; Massie et al., 2011). Cystine is imported and intracellularly converted to cysteine for glutathione (GSH) synthesis (Bannai & Tateishi, 1986; Miura et al., 1992), while glutamate is expelled into the extracellular space (Bannai, 1986). In the genetically engineered xCT knockout (xCT^−/−^) mouse, the START codon for gene *Slc7a11*, which encodes the substrate‐specific light chain of system x_c_^−^ (xCT), is deleted (Sato et al., 1999). Antiporter expression and function are completely undetectable in brain tissue of xCT^−/−^ mice. Although system x_c_^−^ʼs role in GSH synthesis appears vital for cellular health in vitro (Bannai & Tateishi, 1986; Chintala et al., 2005; Shih et al., 2006; Jackman et al., 2010), xCT^−/−^ mice maintain normal brain GSH levels (DeBundel et al., 2011; Sears et al., 2019), thus suggesting auxiliary transporter systems compensate for antiporter loss in vivo (Sosnoski et al., 2020). Remarkably, xCT^−/−^ mice display no behavioural (Featherstone & McCullagh, 2014), neurodevelopmental, or morphological abnormalities despite lacking antiporter expression (DeBundel et al., 2011; Massie et al., 2011).

Because system x_c_^−^ is absent, in vivo microdialysis analyses reveal significantly decreased extracellular glutamate concentrations in hippocampus (DeBundle et et al., al., 2011) and striatum (Massie et al., 2011) of young and old xCT^−/−^ mice—results confirmed by low‐flow push–pull sampling in hippocampal slices (Ojeda‐Torres et al., 2015). Despite the decrement of ambient glutamate, genetic deletion of system x_c_^−^ does not alter hippocampal expression of glial transporters GLT‐1 and GLAST, neuronal transporter EAAC1, or vesicular glutamate transporters VGLUT1–3 (DeBundel et al., 2011). Although whole‐cell patch clamp recordings in juvenile xCT^−/−^ mice suggest an increase in CA1 EPSC amplitudes after stimulation to CA3, this observation is not attendant to alterations in hippocampal cell capacitance, γ‐aminobutyric acid receptor (GABAR)‐ nor NMDAR‐mediated currents (Williams & Featherstone, 2014). Importantly, a recent study found no differences in fEPSP I/O curves at various stimulation intensities between WT and xCT^−/−^ hippocampal slices from adult and aged mice (Verbruggen et al., 2022). That being said, evoked postsynaptic currents are decreased in prefrontal cortex layer II/III (Alcoreza et al., 2019) and striatum (Bentea et al., 2021) in mice after system x_c_^−^ deletion. As such, differences in the I/O relationships of xCT^−/−^ mice may be circuit‐ and age‐dependent.

### 4.2 Post‐hypoxic potentiation in xCT^−/−^ mice shares mechanisms with theta‐burst LTP

The current study utilized a well‐characterized hippocampal slice preparation and experimental design wherein levels of oxygen perfusing in vitro tissue can be precisely controlled (Arrigoni et al., 2005; Coelho et al., 2006; Croning & Haddad, 1998; Dale et al., 2000; Fischer et al., 2009; Heit et al., 2021; Larson & Park, 2009; Lipton, 1999; Pearson & Frenguelli, 2004; Wang et al., 1999). This allows for the appraisal of nuanced alterations in neurotransmission which result from graded changes in ambient oxygen concentrations. Importantly, fluctuations in synaptic transmission induced by mild hypoxia differ from the noxious excitotoxic events triggered by total oxygen deprivation (anoxia). Our experiments concomitantly exposed WT and xCT^−/−^ slices to a bout of mild hypoxia (25% N_2_/75% O_2_) in the same chamber (i.e., paired experiments) while recording neuronal responses from CA1. After baseline measurements, slices were subjected to 30 min of mild hypoxia, reoxygenated and allowed to recover for 45 min. Slices from WT and xCT^−/−^ mice showed equal suppression of synaptic transmission during the hypoxic episode; upon reoxygenation, however, xCT^−/−^ mice exhibited an accelerated rate of recovery and post‐hypoxic potentiation of synaptic transmission when compared to WT controls (Figure 1).

Classical hippocampal LTP, generated by repetitive synaptic stimulation such as TBS, can be characterized as possessing two principal phases: *induction* and *expression*. LTP *induction* requires that the synaptic stimulation sufficiently depolarizes postsynaptic spines to elicit the activation of voltage‐sensitive NMDARs, which allows calcium to penetrate postsynaptic spines (Malenka & Nicoll, 1999). Intracellular calcium then initiates a cascade of biochemical processes requiring calcium/calmodulin‐dependent protein kinase II (CaMKII) (Bliss & Collingridge, 2013). These molecular events trigger increased trafficking and insertion of postsynaptic AMPARs ultimately leading to the *expression* of LTP: an enduring, non‐decremental enhancement of fEPSPs. Our findings suggest that this canonical sequence of events underlies the post‐hypoxic potentiation observed in xCT^−/−^ mice. First, its induction was effectively abolished by the antagonism of NMDARs (Figure 3). Second, experimentation with WT slices showed that the induction and expression of this post‐hypoxic effect required elevated calcium concentrations (Figure 8). Third, the potentiated response was expressed as a stable enhancement of AMPAR‐mediated fEPSPs; on average, the magnitude of this increase reached ≥30% of baseline fEPSPs (Figure 1). Finally, there were no alterations in paired‐pulse facilitation (PPF) throughout the potentiation phase, thus precluding the influence of a presynaptic mechanism (Figure 2). Because of these parallels, we conclude that the post‐hypoxic potentiation observed in xCT^−/−^ slices shares comparable mechanisms to LTP, and we will refer to this phenomenon as ‘post‐hypoxic LTP’. Nevertheless, although post‐hypoxic LTP expression is consistent with a postsynaptic increase in AMPAR expression, we cannot rule out alterations to NMDARs or other receptors.

### 4.3 Post‐hypoxic LTP is not prevented by antagonism of adenosine A_1_ receptors

The dependence of post‐hypoxic LTP on NMDARs suggests that hypoxia itself, or re‐oxygenation after hypoxia, produces a surge of glutamate release which occurs specifically in slices lacking xCT. As such, we examined the role of adenosine, a key regulator of glutamate release during hypoxia, in the post‐hypoxic LTP observed in xCT^−/−^ mice. Prior studies using in vitro hippocampal slices show that mild hypoxic conditions elicit a suppression of synaptic transmission proportional to the degree of oxygen deprivation (Larson & Park, 2009; Larson et al., 2014). The suppression of synaptic transmission during mild hypoxia results not from a direct effect of terminal ATP depletion, but rather from the extracellular accumulation of adenosine released as a consequence of ATP breakdown (Dale et al., 2000; Frenguelli et al., 2003; Laghi Pasini et al., 2000; Heit et al., 2021; Zetterstrom et al., 1982); adenosine binds to presynaptic adenosine A_1_ receptors (A_1_Rs) to suppress glutamate release (Figure 5A). In the early stages of hypoxia, ongoing cellular processes hydrolyse intracellular ATP (to ADP, AMP and adenosine) and its replacement depends on the level of oxygen deprivation (Melani et al., 2012). The intracellular adenosine that builds up is ultimately expelled and accumulates in a time‐dependent manner within the extracellular space (Frenguielli et al., 2007; Jurányi et al., 1999; Melani et al., 2005; Schock et al., 2007). This subsequently inhibits the evoked synaptic glutamate release by activation of pre‐synaptic A_1_Rs (Dunwiddie & Masino, 2001; Fowler, 1989, 1990; Heit et al., 2021; Pearson et al., 2006; Sebastião et al., 2000) and postsynaptic A_1_Rs which purportedly hyperpolarize neurons by opening K^+^ channels (Segal, 1982; Trussell & Jackson, 1987). Synaptic transmission becomes suppressed within the first few minutes of partial hypoxia due to the effects of endogenous adenosine release, and this suppression is entirely reversible upon re‐oxygenation (Fowler, 1989; Heit et al., 2021; Lipton & Whittingham, 1979).

Beyond its acute, hypoxia‐induced suppression of presynaptic glutamate release, extracellular adenosine can modulate neuronal excitability during normoxia via (1) G‐protein‐coupled inhibition of voltage‐dependent calcium channels (VDCCs) (MacDonald et al., 1986), (2) inhibition of spontaneous calcium‐dependent neurotransmitter release (Scanziani et al., 1992), or (3) postsynaptic activation of inwardly‐rectifying K^+^ channels (GIRKS) (Ehrengruber et al., 1997). Most notably, however, endogenous adenosine acting upon A_1_Rs can mitigate the activity of postsynaptic VDCCs and NMDARs (de Mendonca et al., 1995; Klishin et al., 1995) and restrain LTP expression (Arai et al., 1990; Forghani & Krnjevic, 1995; de Mendonça et al., 2000). Considering these A_1_R‐mediated mechanisms, we reasoned that the generation of post‐hypoxic LTP in xCT^−/−^ mice may be due to a reduced effect of adenosine's downstream actions after reoxygenation, or that mutants express lower ubiquitous adenosine levels upon reoxygenation in comparison to WT.

We confirmed that the suppression of glutamate release by hypoxia in both WT and xCT^−/−^ slices was mediated by endogenous adenosine, as the depression of synaptic transmission was eliminated by CPX, an adenosine A_1_R antagonist (Figure 5). In the absence of CPX, the magnitude of synaptic suppression during hypoxia was similar in WT and xCT^−/−^ slices (Figure 1), suggesting that the metabolic effect of hypoxia (net conversion of ATP to adenosine) was unaffected by deletion of xCT. This conclusion was also suggested by an analysis of synaptic suppression early in episodes of anoxia (Heit et al., 2022). Nonetheless, the appearance of post‐hypoxic LTP in xCT^−/−^ mice was not prevented by blocking adenosine A_1_Rs during hypoxia, thus precluding an adenosine‐sensitive mechanism. These findings therefore confirm that (1) post‐hypoxic LTP in xCT^−/−^ slices is not a function of adenosine's neuromodulatory action to decrease glutamate release, (2) A_1_R expression and/or activation is not inhibited in xCT^−/−^ slices as a result of hypoxia, and (3) the magnitude and kinetics of ATP breakdown and adenosine release is unaffected by xCT deletion.

### 4.4 CPG treatment elicits post‐hypoxic LTP in WT mice and attenuates hypoxia‐induced synaptic suppression in both genotypes

To further examine system x_c_^−^ʼs role in post‐hypoxic LTP, we used an antagonist of the antiporter. WT and xCT^−/−^ slices were pretreated with 100 µM CPG for 2 h prior to the hypoxic episode. The drug condition not only caused WT slices to phenocopy xCT^−/−^ slices in the rate of recovery upon reoxygenation, but also generated post‐hypoxic LTP equivalent in magnitude to that of xCT^−/−^ slices (Figure 6) with no changes in PPF (Figure 7). We have previously shown that 2 h of CPG administration elicits a ∼26% increase in baseline AMPAR‐driven fEPSPs in WT slices but not xCT^−/−^ slices (Heit et al., 2022). It is therefore quite striking that hypoxia induces LTP even after the CPG‐mediated enhancement of baseline responses in WT mice. Importantly, the degree of post‐hypoxic LTP in xCT^−/−^ slices in the presence of CPG was equivalent to that attained in its absence (Figures 1 and 6), thus providing strong evidence that the post‐hypoxic LTP in WT slices was due to the inhibition of system x_c_^−^ and not an off‐target drug effect.

Interestingly, the suppression of synaptic transmission during hypoxia was attenuated by CPG in both WT and xCT^−/−^ slices. Accordingly, because both genotypes displayed this response, it is unlikely that the effect was due to the drug's action on system x_c_^−^. Although it is possible that CPG has a direct inhibitory effect on adenosine A_1_Rs, we are unaware of any positive evidence in this regard. That being said, there are indications that mGluR antagonists can mitigate the efficacy of adenosine A_1_R agonists on presynaptic glutamate release (Mendonca & Ribeiro, 1997). CPG has inhibitory effects on both group I and II mGluRs which are heavily expressed in the Schaffer collateral pathway (Hagena et al., 2022). This may explain the attenuation of hypoxia‐induced synaptic suppression in WT and xCT^−/−^ slices; as noted above, suppression was effectively prevented by the A_1_R antagonist, CPX, in both genotypes. All things considered, because WT and xCT^−/−^ slices displayed the same degree of reduced hypoxia‐induced suppression during CPG treatment, the mechanism producing this phenomenon differs from that which evokes post‐hypoxic LTP in CPG‐treated WT mice and is likely an off‐target drug action.

### 4.5 System x_c_^−^ and post‐hypoxic LTP

The present studies employed a mild hypoxia protocol which elicited post‐hypoxic potentiation of synaptic transmission only in conditions of genetic deletion or pharmacological antagonism of system x_c_^−^. More severe oxygen deprivation (anoxia) (Hsu & Huang, 1997) or combined anoxia and aglycaemia (Crépel et al., 1993; Crepel et al., 1993) produce post‐ischaemic effects in rat hippocampal slices that substantially differ from our results. First, the post‐anoxic potentiation in rat slices is expressed solely in responses mediated by NMDARs (Crépel et al., 1993; Crepel et al., 1993) or by responses mediated by both AMPARs and NMDARs (Hsu & Huang, 1997). In the latter case, post‐anoxic potentiation was accompanied by reductions in PPF, thereby indicating a presynaptic expression mechanism. In all those experiments, anoxia triggered AD, an event involving copious glutamate release and potential excitotoxicity. In contrast, the post‐hypoxic LTP described herein was not contingent upon AD, was expressed by AMPAR‐dependent synaptic responses, and did not alter PPF. Furthermore, the post‐hypoxic LTP illustrated in the present study required suppression of the glutamate antiporter, system x_c_^−^, and was prevented by antagonism of NMDARs.

To elucidate our findings in xCT^−/−^ slices, as well as those in CPG‐treated WT slices, we evaluated whether the ratio of Ca^2+^ to Mg^2+^ in the ACSF bath could influence post‐hypoxic LTP (Figure 8). When WT slices were bathed in ACSF containing an equimolar ratio of calcium to magnesium (2 mM:2 mM), EPSPs returned to baseline levels upon reoxygenation. Interestingly, when calcium exceeded magnesium (4 mM:2 mM), post‐hypoxic LTP was evoked in WT slices to the same degree as xCT^−/−^ slices bathed in ACSF containing the equimolar ratio (2 mM:2 mM). In contrast, low calcium conditions produced long‐term depression in WT slices likely due to the decrement of the ion's availability. Thus, incubating WT slices in elevated calcium concentrations revealed that post‐hypoxic LTP can be produced without pharmacological inhibition of system x_c_^−^. Overall, these data indicate that system x_c_^−^ʼs suppressive effect on post‐hypoxic LTP can be overcome by conditions favouring NMDAR‐dependent postsynaptic calcium influx during or after hypoxia.

In our previous study, we showed that the absence or antagonism of system x_c_^−^ mitigates AD and delays its appearance in hippocampal slices exposed to anoxia (Heit et al., 2022). These effects appeared to be due to the antiporter's role in maintaining ambient (tonic) levels of extracellular glutamate. When system x_c_^−^ is absent or inhibited, ambient glutamate is significantly reduced, and AD is delayed and attenuated. It is not obvious how a decrement of ambient glutamate would enable NMDAR‐dependent post‐hypoxic LTP. NMDAR sensitivity implies postsynaptic depolarization and presynaptic glutamate release at levels higher than occur during normal synaptic transmission. There is no evidence that mild hypoxia, as used in the current study, produces sustained postsynaptic depolarization, as moderate oxygen deprivation would be expected to reduce evoked synaptic responses.

Hypoxia equally suppressed synaptic transmission in both WT and xCT^−/−^ slices. This suppression, however, was completely blocked by antagonism of adenosine A_1_Rs via a putative presynaptic mechanism; moreover, A_1_R antagonism did not preclude the generation of post‐hypoxic LTP in xCT^−/−^ mice. It is feasible that the hypoxic insult and/or re‐oxygenation produce(s) an upsurge of glutamate release and postsynaptic depolarization in xCT^−/−^ slices sufficient to relieve the magnesium block from voltage‐sensitive NMDARs. This would consequently allow calcium to penetrate post‐synaptic spines (Lynch et al., 1983) and initiate the biochemical processes (Bliss & Collingridge, 2013) which induce post‐hypoxic LTP. Whether this is dependent upon decreased ambient glutamate due to system x_c_^−^ inactivity or an unknown consequence of the antiporter's function remains unclear. Such an effect might be mediated by a more efficient activation of postsynaptic calcium‐dependent processes necessary for LTP induction.

If mild ischaemia stimulates a rush of glutamate release in xCT^−/−^ mice, it is more likely to occur during reoxygenation as opposed to the hypoxic phase due to the inhibition of glutamate release by adenosine A_1_Rs during hypoxia. Alternatively, the differential post‐hypoxic responses between WT and xCT^−/−^ slices could be explained by variations in NMDA‐dependent postsynaptic calcium influxes or its extrusion. We showed that WT slices incubated in elevated extracellular calcium concentrations (4 mM as opposed to 2 mM) exhibit post‐hypoxic LTP (Figure 8) similar in magnitude to that expressed in xCT^−/−^ slices (Figure 1). These data suggest that the final common pathway for post‐hypoxic LTP in WT slices bathed in ‘high’ extracellular calcium, as well as xCT^−/−^ slices bathed in ‘normal’ extracellular calcium, is postsynaptic calcium influx—as it is for TBS‐induced LTP.

In sum, interference with the operation of system x_c_^−^, either by genetic deletion (Figure 1) or by pharmacological blockade (Figure 6), enabled the generation of post‐hypoxic LTP. Induction of this post‐hypoxic plasticity was contingent upon the activation of NMDARs (Figure 3), but unaffected by antagonism of adenosine A_1_Rs (Figure 5). Because TBS‐induced LTP was not altered by xCT deletion (Figure 4), the data support the hypothesis that inactivity of system x_c_^−^ begets conditions which favour enhanced NMDAR‐dependent calcium build‐up in postsynaptic spines in response to a glutamate surge during re‐oxygenation after hypoxic challenge. Thus, similar to the consequences of TBS‐induced LTP, hypoxia exposure provides a ‘stimulus’ in xCT^−/−^ mice which favours NMDAR activation, thereby allowing calcium influx to drive pathways leading to increased AMPAR expression. As mentioned, these reactions, which culminate as post‐hypoxic LTP, are reproducible in WT slices treated with the system x_c_^−^ inhibitor, CPG. Moreover, the suppressive effect of intact system x_c_^−^ activity on post‐hypoxic LTP can be circumvented when extracellular calcium concentrations are elevated (Figure 8). These collective findings – and their relevance within the broader context of stroke etiology – are schematically summarized in Figure 9.

**Experimental Limitations** Whilst our experiments strongly implicate similar mechanisms for induction of post‐hypoxic LTP and TBS‐induced LTP, whether the long‐term expression mechanisms are identical or not is unclear. We measured a post‐hypoxic potentiation of synaptic transmission mediated by AMPARs that persisted for 45 minutes; however, the maximal duration of this phenomenon would be difficult to assess in vitro. The specificity of the potentiation to AMPARs also remains unknown. Future studies should evaluate post‐hypoxic expression of AMPA and NMDA receptor subunits, as well as the role of postsynaptic cytoskeletal reorganizations, in both WT and xCT^−/−^ mice, as these variables may influence the stabilization of post‐hypoxic LTP. Lastly, it would be of interest to determine if induction of post‐hypoxic LTP occludes that of theta‐burst LTP and vice‐versa.

### 4.6 Neuroplasticity

Although the excitotoxic effects of severe ischaemia are undeniable, mild hypoxia can trigger propitious neuroplastic changes in synaptic morphology, microarchitecture and excitability. For example, brief episodes of ischaemia to in vitro hippocampal slices can increase the number of multiple synapse boutons and perforated synapses (Jourdain et al., 2002), as well as produce dendritic reorganization (Harris et al., 2003; Tanaka et al., 2008) similar to that which is observed after high frequency stimulation (Piccini & Malinow, 2001; Calabresi et al., 2003). Analyses using electron microscopy have also shown increased postsynaptic densities in CA1 rat hippocampal neurons after 15 min of in vivo cerebral ischaemia (Martone et al., 1999). Furthermore, 30 min of hypoxia can alter the phosphorylation state of AMPARs (Lee et al., 2000) and/or increase trafficking and delivery of AMPARs (Whitlock et al., 2006). The time course of these post‐ischaemic synaptic modifications fits within the parameters of our experimentation and may help interpret the present findings.

Importantly, post‐ischaemic increases of excitability in cerebral tissue can facilitate neural repair and recovery (Calabresi et al., 2003; Joy & Carmichael, 2021; Kubis, 2016). For example, functional magnetic resonance imaging activation and cortical excitability in the ipsilesional motor cortex are linearly correlated with motor performance in the 2 weeks following first‐ever stroke in human patients (Du et al., 2018). Additionally, experimental stimulation protocols, when applied to post‐ischaemic tissue, produce prophylactic alterations in the synaptic network. Transcranial magnetic stimulation (TMS), which propagates motor evoked potentials, improves functional outcome via the increase of NMDAR and AMPAR expression in the acute/subacute stages of stroke (Xing et al., 2023). Similarly, optogenetic stimulation to thalamocortical (Tennant et al., 2017) and M1 (Cheng et al., 2014) circuits promotes functional recovery and sensorimotor abilities after stroke in mice, an effect only observed in the post‐ischaemic environment. Furthermore, GABA receptor activity becomes significantly altered after acute ischaemia in rodents (Green et al., 2000), and pharmacological antagonism of tonic GABAergic inhibition in the peri‐infarct region can promote early and enduring muscular recovery (Clarkson et al., 2010). Thus, it is feasible to consider that post‐hypoxic LTP in xCT^−/−^ mice may be enabled by elevated disinhibition.

Although post‐ischaemic potentiation has been postulated as a precursor to irreparable neuronal damage or reperfusion injury, the evidence supports LTP as a crucial mechanism for functional recovery (Di Filippo et al., 2008; Hagemann et al., 1998). Electrically induced LTP in the peri‐infarct cortex of human patients elicits the reorganization of sensorimotor maps and enhances motor performance of affected limbs (Alonso‐Alonso et al., 2007; Di Lazzaro et al., 2010). Moreover, employing TBS protocols with TMS improves upper limb motor function in both subacute (Sánchez‐Cuesta et al., 2023) and chronic (Zhang et al., 2022) stroke patients. Indeed, our data may therefore explain the increased functional recovery and decreased neurological deficits observed after in vivo MCAO in mice (Hsieh et al., 2017) and rats (Domercq et al., 2016) where system x_c_^−^ was inhibited. Post‐hypoxic LTP in conditions of system x_c_^−^ deletion or inhibition may represent one form of increased neuronal excitability, which induces sensorimotor re‐mapping and/or axonal sprouting in the peri‐infarct cortex (Clarkson & Carmichael, 2009). Thus, our findings likely showcase an early indicator of enhanced recovery in the penumbra as opposed to imminent cell damage. As such, it is feasible to consider system x_c_^−^ antagonism as a therapeutic intervention in the days and/or weeks following ischaemic stroke.

### 4.7 Conclusion

Worldwide, stroke remains the leading cause of long‐term disability (Tsao et al., 2023); and yet, historically, efforts to reduce stroke severity have been plagued by translational failure due to gaps in our understanding of cellular mechanisms leading to brain damage after metabolic insult. We conclude that system x_c_^−^ interference is an auspicious target for stroke therapeutics. Pharmacological antagonism or genetic deletion of system x_c_^−^ provides neuroprotection for both the ischaemic core, where glutamate excitotoxicity is mitigated (Heit et al., 2022), and the ischaemic penumbra, where post‐hypoxic LTP is generated, potentially promoting recovery. Indeed, the current data reveal a possible underlying mechanism for improved post‐stroke outcome measures in rodents, where system x_c_^−^ is genetically deleted or inhibited. On a broader scale, we surmise that hypoxia‐induced potentiation via system x_c_^−^ interference is a form of classical NMDA‐dependent LTP with several therapeutic implications. If applied within an adequate clinical window, system x_c_^−^ antagonism may represent one intervention to spur synaptic plasticity toward recovery. Although the mechanisms contributing to the formation of the penumbra remain nebulous, our findings challenge the traditional view which asserts that increased post‐stroke depolarization in penumbral tissue unequivocally begets excitotoxicity (Pietrobon & Moskowitz, 2014; Somjen, 2001). As evidenced herein, increased depolarization, which induces an LTP‐like response after system x_c_^−^ interference, may facilitate functional recovery in the penumbra. As such, our findings portend a potential translational direction as there are currently several FDA‐approved drugs (e.g. Sorafenib™, Sulfasalazine™ and Errastin™) which function as system x_c_^−^ inhibitors.

## AUTHOR CONTRIBUTIONS

All authors have read and approved the final version of this manuscript and agree to be accountable for all aspects of the work in ensuring that questions related to the accuracy or integrity of any part of the work are appropriately investigated and resolved. All persons designated as authors qualify for authorship, and all those who qualify for authorship are listed.

## CONFLICT OF INTEREST

None declared.

## Data Availability

All data reported in this investigation are represented in the manuscript figures and/or are available upon request. Code for the stimulation protocols employed are available upon reasonable request.
